# Antiviral and Cytotoxic Activities of *Ilex aquifolium* Silver Queen in the Context of Chemical Profiling of Two *Ilex* Species

**DOI:** 10.3390/molecules29133231

**Published:** 2024-07-08

**Authors:** Natalia Pachura, Maciej Włodarczyk, Barbara Bażanów, Aleksandra Pogorzelska, Tomasz Gębarowski, Robert Kupczyński, Antoni Szumny

**Affiliations:** 1 Department of Biocatalysis and Food Chemistry, Faculty of Biotechnology and Food Science, Wrocław University of Environmental and Life Sciences, Norwida 25, 50-375 Wroclaw, Poland; 2 Department of Pharmacognosy and Herbal Medicines, Faculty of Pharmacy, Wroclaw Medical University, Borowska 211a, 50-556 Wroclaw, Poland; 3 Department of Pathology, Division of Microbiology, Faculty of Veterinary Medicine, Wroclaw University of Environmental and Life Sciences, 31 C. K. Norwida Street, 50-573 Wroclaw, Poland; 4 Department of Biostructure and Animal Physiology, Faculty of Veterinary Medicine, Wrocław University of Environmental and Life Sciences, 50-375 Wrocław, Poland; tomasz.gebarowski@upwr.edu.pl; 5 Department of Environment, Animal Hygiene and Welfare, Faculty of Biology and Animal Science, Wrocław University of Environmental and Life Sciences, Chełmońskiego 38C, 51-630 Wroclaw, Poland

**Keywords:** *Ilex*, terpenoids, saponins, phenolic acids, antiviral activity, anticancer activity, cytotoxic activity

## Abstract

The leaves of *Ilex paraguariensis* (known as Yerba mate), used as a popular beverage, are a very well-recognized plant material with various biological activities, including analeptic (because of caffeine), anti-obesity (phenolics, saponins), antimicrobial, and antiviral (phenolics, saponins). Here, the chemical compositions of the leaves of two European *Ilex* species (× *meserveae* and *aquifolium*) with three varieties each were investigated. The terpenoid, saponin, and polyphenolic fractions were submitted for LC-MS or GC-MS analysis against a standard Mate leaf. In addition, the aroma profiles of all the species were analysed using HS-SPME-Arrow prior to GC-MS analysis. All fractions were subjected to antiviral and cytotoxic assays. We found 86 compounds in all accessions, with limonene, linalool, and *p*-cymene being predominant. There were minor similarities between the volatile compositions of the European and South American species. We found ursolic and oleanolic acid to be the main compounds in the terpenoid fraction. Mono-caffeoylquinic acids and di-caffeoylquinic acids were the main constituents of the polar fractions. About 180 compounds from the saponin group were tentatively identified, of which 9 and 3 were selected as distinctive markers for *I. meserveae* and *I. aquifolium*, respectively. Based on chemical screening, *I. aquifolium* Silver Queen was chosen as the source of terpenoid and saponin fractions and polyphenol extracts. The most substantial inhibition of cancer cell growth was observed with saponin in the case of the MCF7 (human breast cancer) cell line, while for LoVo and L929 cell lines (human colorectal cancer and reference mouse fibroblasts), it was slightly weaker. These results should be analysed further as a promising chemoprevention of colorectal and gastrointestinal cancers. Saponin and polyphenolic extracts exhibited similar activities against HSV-1 and HAdV-5, with 4-log reduction in virus titres. This study focuses our attention on a field of potential antiviral formulations derived from European holly.

## 1. Introduction

Plants of the *Ilex* species are used in both pharmaceutical and food industries. Among the best-known and -recognized is the South American *I. paraguariensis*. It is commonly used as an infusion (Yerba Mate) and has primarily stimulating properties due to its caffeine content [1,2]. Additionally, infusions of *I. paraguariensis* have been documented to have anti-cholesterol and antidiabetic effects [3]. Regular consumption of Yerba Mate improves body weight composition, appetite, and mood. Water extracts of this plant have a proven cytotoxic activity against several cancer lines, including colorectal [4], colon [5], liver, and colorectal adenocarcinoma (in the rat model) [6]. The ethyl acetate fraction of *I. paraguariensis* inhibits *Herpes simplex* virus Type 1 and Type 2 replication [7]. Polyphenol-rich extracts of *I. paraguariensis* inhibited 3CL protease in SARS-CoV-2 [8,9].

The biological activities of both extracts and fractions of European taxons of *Ilex* such as *I. aquifolium* Aurea Marginata, Silver Queen, Handsworth New Silver; and *I. meserveae* Blue Prince, Blue Girl, and Blue Princess were analysed. Our previous research showed excellent anti-cholesterol and hepatoprotective activities of water extracts of *I. aquifolium* in Wistar rats [10]. Additionally, its terpenoid and saponin fractions positively affected metabolic parameters through the inhibition of ACAT-1 gene expression in the liver of Zucker rats (fa/fa); this can be helpful in the prevention of metabolic disorders [11]. Caffeoylquinic acids isolated from *I. pubescens* (native to China) strongly inhibited neuraminidase of Influenza A virus by binding to Tyr100, Gln412, and Arg419, leading to inhibition of replication [12]. In addition, 3,4-di-*O*-caffeoylquinic acids isolated from *I. kaushue* revealed activity in the initial phase of enterovirus A71 infection by directly targeting the virus particle and disrupting its attachment to host cells [13]. Saponins such as asprellosides isolated from *I. asperella* inhibited the replication of influenza A virus by up to 63%, with an IC_50_ of approximately 9 µm [14]. Molecular docking simulations suggest that the mechanism of inhibition of triterpenoid compounds involves binding to hemagglutinin.

This study aimed to verify whether easily available European holly species (*I. aquifolium* and *I. meserveae*) also exhibit antiviral activity against enveloped and non-enveloped human viruses (human herpesvirus 1 and adenovirus 5, respectively). To test this, one non-polar (terpenoid) and two polar fractions (polyphenols + phenolic acids and saponins) of leaf extracts from three varieties of two European *Ilex* species, *I. aquifolium* and *I. meservae*, were characterized chemically and compared to I. paraguariensis. We decided to perform a detailed GC-MS characterization of the non-polar fraction together with LC-MS/MS profiling of the saponins because based on our previous experience, they play a key role in the biological activity of European taxons of *Ilex* [10,11]. Ahead of the possible use of European holly leaves as food supplements, a biological study was carried out using normal and cancer cell lines. Additionally, microextraction of volatile organic compounds (VOCs) in the solid phase (HS-SPME-Arrow) revealed the components responsible for the aroma of the studied *Ilex* cultivar leaves. These can play a key role in the sensory properties of developed water infusions.

## 2. Results and Discussion

### 2.1. Volatile Organic Compounds in Ilex

The present study compares the European varieties *Ilex aquifolium* and *Ilex meserveae* with the South American variety *Ilex paraguariensis*. A comparison of the profiles of volatile compounds of European and Argentinian taxons was reported for the first time in our previous publication [11]; however, the VOC profile shown in Table 1 is not identical to the profile obtained in our last paper. This may be due to using the SPME-Arrow technique, which has higher efficiency and sorption volume, especially in complex matrices, as other researchers have shown [15,16]. During the analysis of the profiles of volatile compounds, 86 compounds were identified; the 15 key ones are presented in Table 1 (all the compounds are presented in Table S1). The results presented in Table 1 show similarities in qualitative but not quantitative compositions.

**Figure 1:**
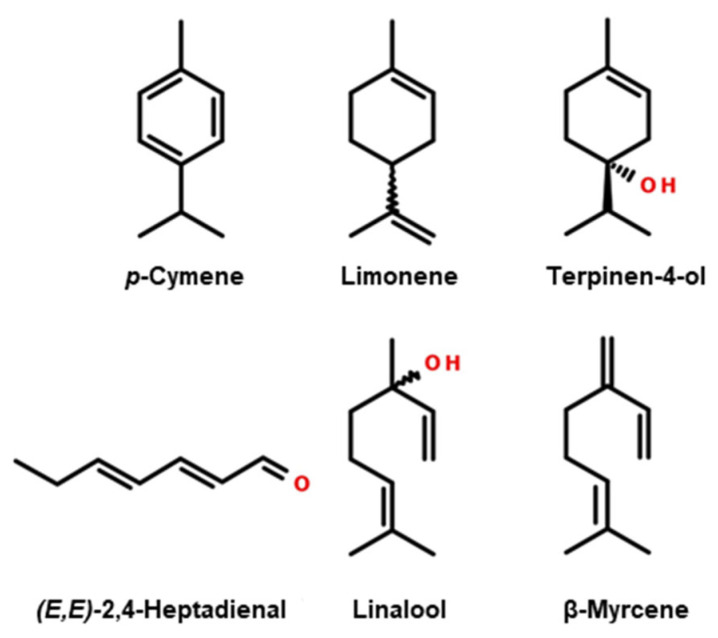
Structural formulas of the main compounds in the aromatic profiles of leaves of *I. paraguariensis* and European cultivars of *I. aquifolium* and *I. meserveae*.

**Figure 2:**
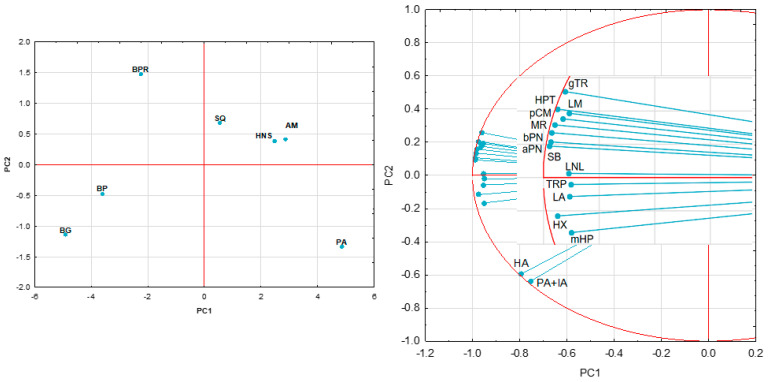
Principal component analysis of *Ilex* taxons (PC1 vs. PC2) based on the contents of its volatile organic constituents; AM—*I. aquifolium* Aurea Marginata, BG—*I. meserveae* Blue Girl, BP—*I. meserveae* Blue Prince, BPR—*I. meserveae* Blue Princess, HNS—*I. aquifolium* Handsworth New Silver, PA—*I. paraguariensis*, SQ—*I. aquifolium* Silver Queen; aPN—α-pinene, bPN—β-pinene, gTR—γ-terpinene, HA—hexanoic acid, HPT—(*E*,*E*)-2,4-heptadienal, HX—hexanal, LA—linalyl acetate, LM—limonene, LNL—linalool, mHP—6-methyl-hept-5-en-2-one, MR—myrcene, PA+IA—pentanoic acid + isoamyl acetate, pCM—*p*-cymene, SB—sabinene, TRP—terpinen-4-ol.

The main compounds (whose chemical structures are shown in Figure 1) identified in the aromatic profile of *I. paraguariensis* leaves were hexanoic acid, *p*-cymene, and limonene, while those in the varieties of European origin were linalool, *p*-cymene, and limonene. The contents of *p*-cymene and limonene in European varieties are significantly higher than in Yerba Mate. Furthermore, an approximately four-fold increase in the *p*-cymene content was observed in some varieties of *I. meserveae* compared with the Mate sample. Significantly higher hexanal, α-pinene, and β-pinene contents were observed in *I. aquifolium* and *I. meserveae* varieties. Figure 2, illustrating the results of PCA, showed that the first two PCs explained over 96% of the variability present in the data, with PC1 explaining 89.21% of the variation, thus allowing the samples to be differentiated. *I. paraguariensis* and *I. aquifolium* had strong positive PC1 scores, whereas the *I. meserveae* varieties were on the negative side of PC1. The *I. aquifolium* samples were quite similar and clustered close together in the middle of the graph. The loading plot for volatile compounds showed strong positive correlations between almost all the compounds identified in Table 1.

Marquez and his team previously showed that the key aroma compounds in *I. paraguariensis* leaves were hexadecane, linalool, geranyl acetone, and (*E*,*E*)-2,4-heptadienal [17], while Kaltbach et al. proved that the key compounds in Yerba Mate were linalool, eucalyptol, limonene, decanal, and pinene [18].

### 2.2. Higher Terpenoids and Sterols

**Figure 3:**
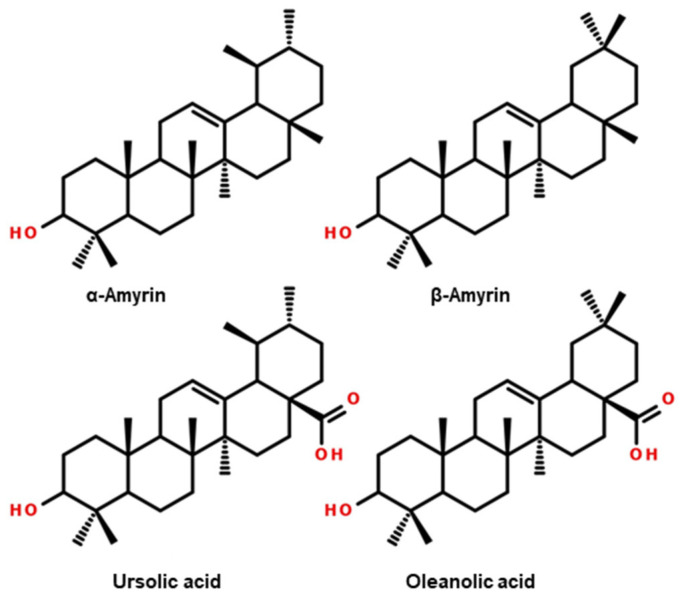
Structural formulas of the main higher terpenoids and sterols in the extracts of leaves of *I. paraguariensis* and European cultivars of *I. aquifolium* and *I. meserveae*.

Analysis of the profiles of higher terpenoids and sterols in *Ilex* leaves showed the presence of 11 compounds (presented in Table 2). For the European cultivars, the study showed similarities in the terpenoid profiles. The dominant compounds (whose chemical structures are shown in Figure 3) in *I. aquifolium* and *I. meserveae* were ursolic acid (10.7–13.3 mg g^−1^), oleanolic acid (3.1–5.1 mg g^−1^), α-amyrin (0.7–2.6 mg g^−1^), lupeol (0.2–0.8 mg g^−1^), and uvaol (0.2–0.7 mg g^−1^). Compared to the European varieties, the leaves of *I. paraguariensis*, which were used as a reference sample, had significantly lower contents of the compounds mentioned above. In particular, an approximately 3× lower content of oleanolic acid (1.54 mg g^−1^) and about 2× lower content of α-amyrin (0.4 mg g^−1^) were identified.

**Figure 4:**
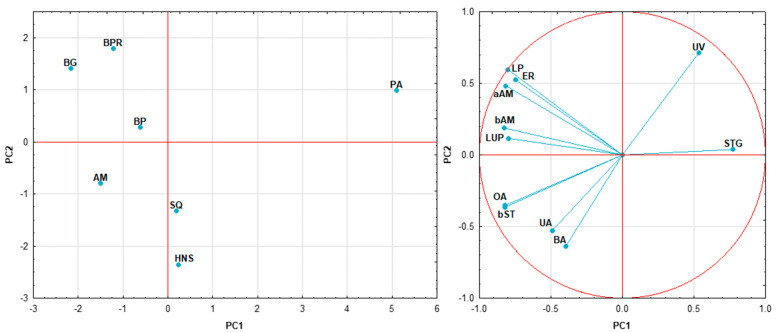
Principal component analysis of *Ilex* taxons (PC1 vs. PC2) based on the contents of its higher terpenoid constituents; AM—*I. aquifolium* Aurea Marginata, BG—*I. meserveae* Blue Girl, BP—*I. meserveae* Blue Prince, BPR—*I. meserveae* Blue Princess, HNS—*I. aquifolium* Handsworth New Silver, PA—*I. paraguariensis*, SQ—*I. aquifolium* Silver Queen; aAM—α-amyrin, BA—betulinic acid, bAM—β-amyrin, bST—β-sitosterol, ER—erythrodiol, LP—lupeol, LUP—lup-20(29)-ene, OA—oleanolic acid, STG—stigmasterol, UA—ursolic acid, UV—uvaol.

Figure 4 shows the results of PCA of triterpenoids found in Ilex. Both components explained ~74% of the variation, with the first one achieving a score of 52.88%. The plot enables the distinguishment of *I. paraguariensis*, which has strong positive PC1 and PC2 scores, whereas the European varieties are in opposition and are located in the central part of the plot. Almost all compounds, except uvaol and stigmasterol, have negative loadings in PC1. Compounds such as lupeol, erythrodiol, α-amyrin, β-amyrin, lup-20(29)-ene, and others located on the upper left quarter of the graph are positively correlated with *I. meserveae* samples. In contrast, triterpenic acids are associated with *I. aquifolium*. The compounds listed above showed no correlation with uvaol. Stigmasterol showed a negative correlation with lupeol and α-amyrin.

For *I. paraguariensis* leaves, Mateos et al. used the HPLC-DAD to demonstrate that the contents of ursolic acid and oleanolic acid, as aglycones, are 1.56–1.81 mg g^−1^ and 0.23–0.31 mg g^−1^, respectively [19]. Reports from other researchers show that higher terpenoids have also been confirmed in other varieties of *Ilex*. Claiton, in his research on *Ilex chamaedryfolia*, isolated and confirmed the structure of α-amyrin and ursolic acid, in addition to isolating three saponin compounds whose structures were confirmed spectroscopically as well as using mass spectrometry [20]. Another species, *Ilex spinigera*, which was researched by an Iranian team, yielded β-amyrin, lupeol, lanosterol, taraxasterol, and moretenol [21].

Meanwhile, Brazilian researchers studying organic extracts from *Ilex guayusa* leaves found that one of the main constituents, besides caffeine and squalene, was α-amyrin, which had a content of up to 0.4 mg g^−1^ depending on the extraction method used [22]. Furthermore, in the *Ilex buxifolia* and *Ilex affinis* varieties studied by Taketa et al., ursolic acid and its derivatives, uvaol, and triterpenoid glycosides were isolated [23]. Another study by the same researchers also isolated ursolic acid as a predominant compound in *Ilex brevicuspis* leaf fractions [24]. The results obtained by our team and teams from different parts of the world confirm the structural specificity of triterpenoids in plants in the *Ilex* taxon.

### 2.3. Phenolic Acids and Polyphenols

**Figure 5:**
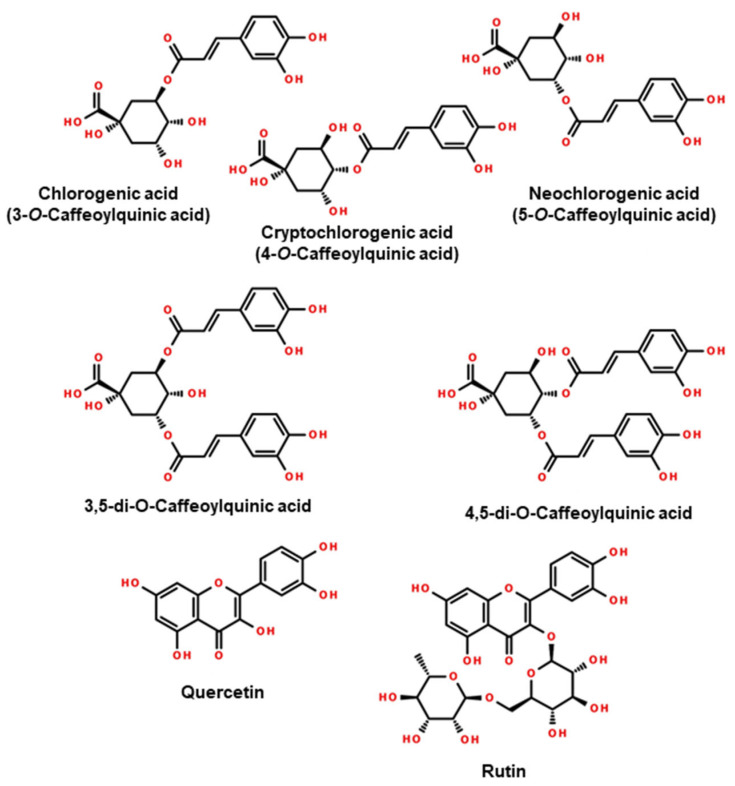
Structural formulas of the main compounds in the phenolic acids and polyphenols profiles of leaves of *I. paraguariensis* and European cultivars of *I. aquifolium* and *I. meserveae*.

Our research on phenolic acid and polyphenol fractions identified 12 compounds (Table 3). Quantitative differences in content were demonstrated between European varieties of *I. aquifolium* and *I. meserveae* and the Argentinian variety *I. paraguariensis*. For the world-famous Yerba Mate, the compounds with the highest content were 3,5-di-*O*-caffeoylquinic acid (18.1 mg g^−1^), chlorogenic acid (13.5 mg g^−1^), neochlorogenic acid (7.5 mg g^−1^), and 4,5-di-*O*-caffeoylquinic acid (6.1 mg g^−1^). European varieties, on the other hand, had significantly higher contents of chlorogenic acid (17.5–39.0 mg g^−1^), neochlorogenic acid (5.7–21.6 mg g^−1^), cryptochlorogenic acid (3.8–9.5 mg g^−1^), and rutin (5.1–11.8 mg g^−1^). On the other hand, these varieties had lower contents of 3,5-di-*O*-caffeoylquinic acid (1.3–7.4 mg g^−1^) and 4,5-di-*O*-caffeoylquinic acid (0.6–3.4 mg g^−1^). The chemical structures of the major compounds are shown in Figure 5.

**Figure 6:**
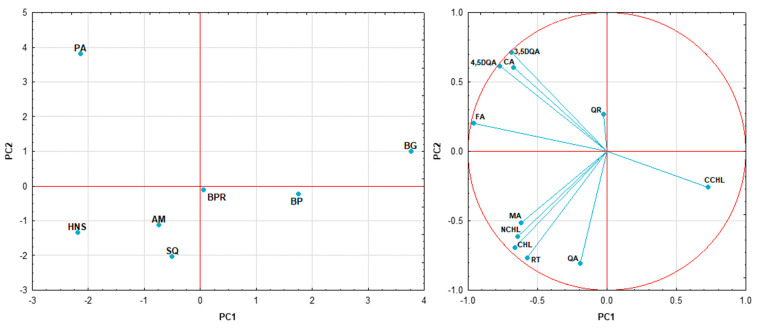
Principal component analysis of *Ilex* taxons based on the contents of its polyphenol and phenolic acid constituents; PA—*I. paraguariensis*, AM—*I. aquifolium* Aurea Marginata, SQ—*I. aquifolium* Silver Queen, HNS—*I. aquifolium* Handsworth New Silver, BP—*I. meserveae* Blue Prince, BG—*I. meserveae* Blue Girl, BPR—*I. meserveae* Blue Princess; RT—rutin, QA—quinic acid, FA—ferulic acid, CA—citric acid, MA—malic acid, QR—quercetin, CCHL—cryptochlorogenic acid, NCHL—neochlorogenic acid, CHL—chlorogenic acid, 3,5DQA—3,5-di-*O*-caffeoylquinic acid, 4,5DQA—4,5-di-*O*-caffeoylquinic acid.

PCA of polyphenols and phenolic acids was carried out, as shown in Figure 6. PC1 and PC2 explain ca. 76% of the variation; therefore, the data were divided into three clusters on the graph. The right cluster, located on the positive side of PC1, contains three varieties of *I. meserveae*, while the left cluster on the negative side includes *I. aquifolium* and the reference samples. On the other hand, *I. paraguariensis* is the outlier, with its PC2 score (about 4) being significantly higher than the others. The loading plot shows two groups of strong positive correlations: the 1st group consists of ferulic acid, 3,5-di-*O*-caffeoylquinic acid, 4,5-di-*O*-caffeoylquinic acid, and citric acid, which are correlated with the reference Mate leaf, and the 2nd group contains malic acid, neochlorogenic acid, chlorogenic acid, and rutin, which are positively correlated with all *I. aquifolium*. In addition, cryptochlorogenic acid seems to be able to distinguish *I. meserveae*.

Many studies have been conducted on phenolic acid and polyphenol fractions from both *I. paraguariensis* and other *Ilex* taxa. A Polish team also conducted research on bioactive compounds in Yerba Mate, which is available on the local market. Quantitative analysis showed the presence of chlorogenic acids, with neochlorogenic acid as the dominant (5.4–39.0 mg g^−1^), followed by chlorogenic acid (1.8–19.0 mg g^−1^) and cryptochlorogenic acid (3.5–17.8 mg g^−1^). This shows a different trend from that observed in our study. In contrast, the caffeic acid content was similar (0.4–0.6 mg g^−1^) [25]. In their study on the contents of flavonoids, phenolic acids, and xanthines in Yerba Mate, Bojic et al. found that the contents of chlorogenic acid and rutin in the phenolic acid fraction were 21.6 mg g^−1^ and 6.8 mg g^−1^, respectively [26]. Simultaneously, Berte et al. conducted a study of the contents of bioactive compounds in spray-dried extracts and dehydrated leaves of *I. paraguariensis.* The contents of rutin (5.4 mg g^−1^), caffeic acid (1.5 mg g^−1^), and neochlorogenic acid (91.4 mg g^−1^) were significantly higher than in dehydrated leaves, which contained 3.1 mg g^−1^, 0.7 mg g^−1^, and 24.8 mg g^−1^ [27].

A study by Paluch et al. determined the contents of chlorogenic acids in European varieties of *I. aquifolium* Argentea Marginata and *I. meserveae* Blue Angel, and identified chlorogenic acid as the predominant compound (11.6–23.3 mg g^−1^), with neochlorogenic (1.7–5.1 mg g^−1^) and cryptochlorogenic (2.2–5.3 mg g^−1^) being present as minor isomers.

However, in *I. paraguariensis*, neochlorogenic acid (19.7 mg g^−1^) was present at the highest concentration. Quantitatively, 3,5- and 4,5-di-*O*-caffeoylquinic acids in both European and Argentinian varieties were present at levels comparable to our results. The rutin content in the samples from the Argentinian species was about half of that in European *Ilex*, which is also confirmed in Table 3 above [28]. Zhu et al. obtained a lower rutin content of about 0.8 mg g^−1^ and a chlorogenic acid content of 0.3–28.0 mg g^−1^ from the Chinese *Ilex kudingcha* and *Ilex cornuta* species [29].

Kelebek et al. observed a relationship analogous to that observed by our team when they analysed chlorogenic acids (chlorogenic acid > neochlorogenic acid > cryptochlorogenic acid) in water extracts of *Ilex guayusa*. However, the quantitative relationship between 3,5 and 4,5-di-*O*-caffeoylquinic acids was the opposite; that is, 2.3 g L^−1^ and 3.4 g L^−1^ [30]. Using LC-MS/MS, a team of researchers qualitatively identified the chlorogenic acids, 3,5 and 4,5-di-*O*-caffeoylquinic acid, caffeic acid and vanillic acid, and the glucosides of the aforementioned phenols in *I. glabra* [31]. Negrin’s team from the USA tested more than a dozen *Ilex* samples for metabolomic and chemotaxonomic purposes. Compared to *I. paraguariensis*, where the total chlorogenic acid content was 45.0 mg g^−1^, the only one where the content was higher was *Ilex cause* (over 50.0 mg g^−1^). The total contents of this compound in *I. guayusa*, *I. vomitoria*, *I. meserveae,* and *I. myrtifolia* were similar to that of Yerba Mate. Among the species where the content of chlorogenic acids was less than 10 mg g^−1^ were *I. crenata*, *I. asprella,* and *I. ambigua* [32]. Chlorogenic acid contents of 0.92% and 0.19%, 3,5-di-*O*-caffeoylquinic acid contents of 0.4% and 0.1%, and 4,5-di-*O*-caffeoylquinic acid contents of 0.5% and 0.2% were identified in *I. brevicuspis* and *I. pseudobuxus*, respectively, while the contents of rutin were 0.06% and 0.03% [33].

### 2.4. Saponins

LC-HRMS and LC-MS/MS mapping of saponins in 70% methanol extracts of the leaves of our *I. aquifolium* and *I. meserveae* samples against a reference sample of Yerba Mate identified about 180 peaks as highly probable saponins. Due to the high number of components and numerous possibilities of identifying similar substances, each was named using an acronym containing its pseudomolecular ion and the averaged retention time under experimental conditions (e.g., 911, 12.60 min).

Summarising the extensive results presented in Supplementary Table S2, 10 saponins with RA% higher than 75% were recorded. Among them, the main saponins, clearly differentiating *I. aquifolium* and *I. meserveae* samples from *I. paraguariensis* based on their relative concentrations, were (1073, 8.11 min), (927, 8.29 min), (911, 12.6 min), and (765, 15.25 min), as well as one standout compound containing the SO_3_ group (887-S, 10.71 min). Another 27 saponins with RA% higher than 25% were recorded.

For the group of *I. aquifolium* cultivars, the distinctive saponin markers may be the following three compounds: (969, 9.07 min), (969, 9.5 min), and (887-S, 10.71 min).

For the group of *I. meserveae* cultivars, the distinctive saponin markers may be the following nine compounds: (927, 7.07 min), (1235.61, 7.51 min), (825, 7.71 min), (809, 8.11 min), (793, 12.6 min), (911, 13.98 min), (911, 14.25 min), (765, 15.25 min), and (647, 16.50 min).

For the reference (PA), the most outstanding saponins were (809, 9.07 min), (809, 9.27 min), (1115, 11.32 min), (777, 11.5 min), (1057, 12.18 min), (911, 12.6 min), (895, 14.43 min), and (953, 14.96 min).

According to the results of the GC analysis of the terpenoid fraction (reported above), the main aglycones of saponins in the investigated *Ilex* taxons are ursolic and oleanolic acids.

**Figure 7:**
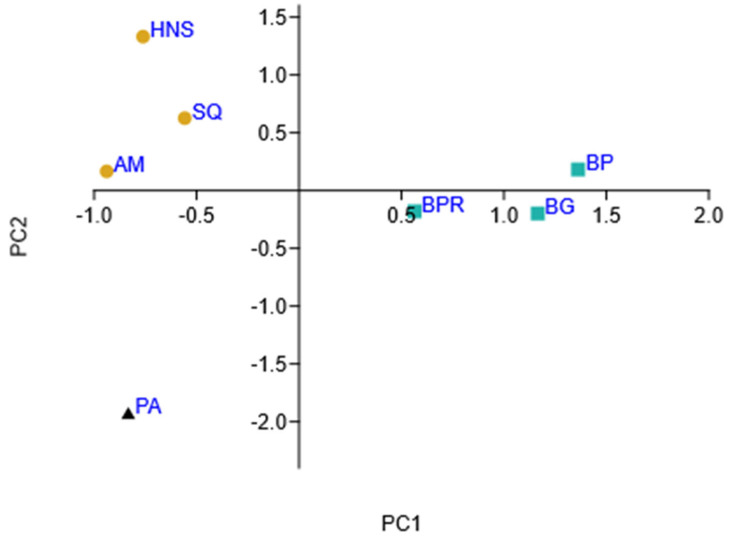
Principal component analysis of *Ilex* taxons (PC1 vs. PC2) based on saponin content; AM—*I. aquifolium* Aurea Marginata, BG—*I. meserveae* Blue Girl, BP—*I. meserveae* Blue Prince, BPR—*I. meserveae* Blue Princess, HNS—*I. aquifolium* Handsworth New Silver, PA—*I. paraguariensis*, SQ—*I. aquifolium* Silver Queen.

Similarities and differences between the saponin patterns of the analysed *Ilex* samples were visualised using hierarchical clustering (Supplementary Materials Figure S1) and principal component analysis (Figure 7). PCA explained about 62% of the variation, and the graph shows three groups in different locations. The *I. paraguariensis* reference sample has strong negative PC1 and PC2 scores, while the three varieties of *I. meserveae* are located on the positive side of PC1. In contrast, *I. aquifolium* varieties are located in the upper left quarter of the graph (negative PC1 and positive PC2 scores).

### 2.5. Antiviral Properties

The three fractions of *I. aquifolium* Silver Queen (polyphenols, saponins, and higher terpenoids) were tested for antiviral properties against HSV-1 and HAdV-5 (Table 4). It was observed that the degree of reduction of replication of both viruses by polyphenols and saponins was 4 log (99.99% reduction). For the terpenoid fraction, a similar efficacy (99.99%) was observed against HSV-1, while the reduction level was 2 log (99% virucidal efficacy) against HAdV-5 [34,35].

A parallel evaluation of the cytotoxicity of the formulations at the concentrations used in the virucidal tests showed no changes in the cultured cells.

The exact mechanism of the observed action of the tested extracts and fractions is not yet known. Presumably, it is based on the action of a nonpolar (terpenoid) and a saponin fraction. Terpenoids are a diverse class of phytochemicals derived from isoprene units, which numerous authors have reported to show significant potential as antiviral agents against a broad spectrum of enveloped and nonenveloped viruses [36]. According to Xu et al., they interfere with virus entry into the host cell in the early stages of infection, which hinders its replication [37] and modulates the host’s immune responses. For example, anti-HIV effects are believed to include activation of protein kinase C isoenzymes [38]. Based on the tests carried out in this project, the substances in the fractions have virucidal potential, probably linked to blocking or destroying the virus proteins, which prevents them from attaching to the cell, although we cannot exclude a parallel antiviral effect. This type of multidirectional action of *Garcinia parvifolia* leaf extract was demonstrated in a study by Adnan et al. [39].

Their multifaceted mechanisms of action make them attractive agents for combating viral infections and offer opportunities for synergy with existing antiviral drugs. So far, they have been confirmed to be effective against influenza viruses, SARS-CoV-2, HCV (hepatitis C), and HSV, among others [36]. Furthermore, according to a study analysing HSV-1 therapy using simultaneous administration of acyclovir and betulin, these substances were found to have a strong synergistic effect against HSV [40]. However, further research is needed to clarify the mechanisms of action of *Ilex* terpenoids against different viruses. According to Orosco and Quimque, terpenoids showed potent antiviral activity against a wide range of human and animal viruses. Their diverse structural and chemical properties offer promising opportunities for the development of novel antiviral agents [36].

The second group of compounds in *Ilex* extracts is saponins, which have a variety of biological activities, including antiviral activity. In a study by Simões et al., two substances isolated from natural sources due to activities associated with their amphiphilic constituents were tested for their effects on the replication of herpes simplex virus type 1. A triterpenoid saponin representing the oleanane group inhibited viral DNA synthesis, while a triterpenoid saponin representing the ursane group inhibited viral HSV-1 capsid protein synthesis [41]. The antiviral activity of saponins has also been proven against other viruses such as influenza virus, human respiratory syncytial virus (RSV), and SARS-CoV-2. The mechanisms of antiviral action, as with terpenoids, depend both on the substance itself and on the virus; they include interaction with and destruction of viral envelope proteins, prevention of virus binding to host cells by damaging viral binding sites, and coating of viral receptors on host cells [41].

### 2.6. Anticancer Properties

The chemopreventive activities of PLHA extract (polyphenols and phenolic acids), SAP extract (saponins), and TERP extract (terpenoids) were assessed using the cell culture model. The SRB assay, which measures the cytotoxic effect by measuring the amount of protein, was chosen for the biological activity tests. The choice of assay was associated with the characteristics of extracts tested and the possibility of errors in assays measuring mitochondrial activity.

As a result of the study, a GI50 (cell growth inhibition) dose was determined for the extracts tested, determining a dose that inhibits the growth of individual cell types by 50%.

Table 5 shows the results of the inhibition of tumour cell growth. The promising results indicate the high chemopreventive activity of the PLHA and SAP extracts. The results showed weak growth inhibitory activity against normal cells and high activity against four tumour cell lines. In the case of L929, a mouse cell line, the SAP fraction also showed growth inhibition; however, this is a rapidly proliferating line. In the case of the NHDF cell line, such an effect was not found. The results indicate a potential effect on the prevention of colorectal cancer. Chlorogenic acids and saponins are usually not very well absorbed from the intestines; thus, the gut can be the target of their preventive action. Moreover, because a group of willing Mate drinkers should be easy to find, the population susceptibility to various intestinal cancers can be examined against reluctant Mate drinkers to find any correlation. The dietary use of such extracts, which limit cell growth, affects the first and most important stage of carcinogenesis. In the case of the A549 and MCF7 cell lines, the potential beneficial therapeutic effects will depend on the absorption and distribution of the compounds in the body. The observed activity raises hopes for the broad applicability of the tested extracts in chemoprevention, in addition to cases of cancers not located in the gastrointestinal tract.

We focused on the notable safety index between the concentrations that inhibit tumour and normal cell growth. In more difficult cases (HT29/NHDF), it was about 1:7 for PLHA and 1:22 for SAP, while some cancer lines were even more selectively susceptible to the tested mixtures (for LoVo/NHDF it was about 1:30 for PLHA and 1:50 for SAP). The greater the second value in the above ratios, the safer the tested extracts are for the patient.

The results presented in Table 6 indicate selective tumour cell growth inhibitory activity against fibroblast lineages. Selective growth inhibition is very intuitive for compounds with chemopreventive activity, as even low doses can limit the growth of tumour cells. SAP extract seems to be particularly significant in terms of activity.

Figures S2 and S3 (Supplementary Materials) show the results of the SRB test depending on the concentration of the tested extracts. Figure S2 shows the results for the mouse cell line L929 and normal human dermal fibroblast lines. Both lines were standardly used to assess the cytotoxicity of new compounds. In the case of the NHDF cell line, all extracts tested had low activity. In the case of the L929 cell line, a strong growth inhibitory effect was found for SAP; however, there was no cytotoxic effect. Moderate inhibition of normal cell growth may have a beneficial chemopreventive effect on the first stage of the development of the tumour, with cells proliferating excessively, or in the case of wound healing, when there is a problem with excessive wound granulation.

Figure S3 shows the extract’s effect on the growth of cells in tumour cell lines. In this case, both a cytostatic and a cytotoxic effect are evident. The effect of the tested extract is dose-dependent. For SAP and PLHA extracts, anticancer activity was apparent at the lowest extract doses. This is significant for their use in the chemoprevention of gastrointestinal cancers.

Although the plant under study is well-known, information on chemopreventive activity is scarce. The chemopreventive activity of *I. paraguariensis* in a cell culture model was confirmed using the HepG2 cell line [42]. Confirmation of chemopreventive activity has also been presented in other publications, although these are few [43,44].

## 3. Materials and Methods

### 3.1. Plant Material

The European *Ilex* varieties were supplied by Henryk Różański, PhD, from his own cultivation (Krosno, Poland 49°40′22′′ N 21°46′06′′ E). The shrubs were grown in medium loamy humus soil. The plants (*I. aquifolium* Aurea Marginata—AM, *I. aquifolium* Silver Queen—SQ, *I. aquifolium* Handsworth New Silver—HNS, *I. meserveae* Blue Prince—BP, *I. meserveae* Blue Girl—BG, *I. meserveae* Blue Princess—BPR) were harvested in September 2023. The leaves were separated from the stems and the dust was cleaned off. They were then freeze-dried for 24 h using a Lyovac GT 2 apparatus. The dried material (approximately 400 g) was ground to homogenise it for further study. The reference for the survey was a sample of *I. paraguariensis* dried leaves (abbreviated PA; harvested in Argentina in 2022) purchased from a local distributor. Specimens of European varieties of *Ilex* are deposited in a local herbarium. 

### 3.2. HS-SPME-Arrow-GC-MS

Powdered *Ilex* leaves (<0.05 mm) weighing 200 mg and 0.5 µg of 2-undecanone (Sigma-Aldrich, Steinheim, Germany) as an internal standard were placed in a headspace vial. Analyses were performed on a Shimadzu GCMS QP 2020 Plus (Shimadzu, Kyoto, Japan) equipped with a Zebron ZB-5 MSi capillary column (30 m × 0.25 mm × 0.25 μm; Phenomenex, Torrance, CA, USA). A 1.10 mm DVB/C-WR/PDMS SPME Arrow fibre (Shimadzu, Kyoto, Japan) was used for extraction. Before extraction, the samples were conditioned for 10 min at 50 °C. The desorption of volatile compounds was carried out in an injector at 250 °C and lasted 3 min (split ratio; helium was used as the carrier gas, with a column flow of 1 mL min^−1^). The GC oven gradient was programmed as follows: start at 50 °C, then increase to 130 °C at a rate of 4 °C min^−1^, then to 180 °C at a rate of 10 °C min^−1^, and finally to 280 °C at a rate of 20 °C min^−1^. The MS operating conditions were as follows: ion source temperature of 250 °C, interference temperature of 250 °C, and scanning at 40–400 *m*/*z*. The analysis was performed in triplicate.

The results were evaluated by comparing experimentally determined retention indices calculated against n-alkane mixtures (C_7_–C_40_, Sigma-Aldrich, Steinheim, Germany) with retention indices and mass spectra available in the Flavours and Fragrances of Natural and Synthetic Compounds 3.0 (FFNSC 3.0) database and the NIST23 library. The search filters were set to achieve mass spectrum similarities ≥90% and RI ± 10. Chromatograms were analysed using ACD/Labs Spectrus 2021.2.1 software (Toronto, ON, Canada).

### 3.3. Higher Terpenoids and Sterols

Dry powdered *Ilex* leaves weighing 50 g (<0.05 mm) were macerated twice for 24 h at room temperature with acidified ethanol (pH = 3, using HCl). For GC-MS analysis, an internal standard of cholesterol was added to each sample. The macerates were filtered, combined, centrifuged (5 min 13,000 rpm), and concentrated using a vacuum evaporator. The residue was extracted three times with 20 mL of dichloromethane and washed with saturated aqueous NaCl solution. The entire mixture was centrifuged again (5 min 13,000 rpm) and the organic layer was transferred to a round bottom flask and evaporated until dry. The fraction prepared in this way was then used for biological assays by getting the extract to the appropriate concentration in dedicated solvents (see Section 3.6 and Section 3.7 for information). The profile of higher terpenoids and sterols was identified using the *N*,*O*-bis-(trimethylsilyl)trifluoroacetamide (BSTFA) derivatization method [10]. For this purpose, 500 µL of pyridine and 50 µL of BSTFA were added to the sample and the mixture was transferred to a vial and heated at 70 °C for 15 min. The analysis was performed in triplicate.

The analyses were performed on a Shimadzu GCMS QP 2020 Plus instrument (Shimadzu, Kyoto, Japan) equipped with a Zebron ZB-5 MSi capillary column (30 m × 0.25 mm × 0.25 μm; Phenomenex, Torrance, CA, USA). GC-MS operating conditions were as follows: injector temperature 280 °C, split ratio 10, and column flow 0.95 mL min^−1^. Scanning was performed from 40 to 720 *m*/*z* using electron impact ionisation (EI) at 70 eV. The GC oven was programmed with helium as a carrier gas as follows: initial temperature 180 °C held for 1 min, then increased at a rate of 2 °C min^−1^ to 300 °C and held for 10 min.

Identification of the obtained analytes was performed using the same procedure as in Section 3.2.

### 3.4. Phenolic Acids and Polyphenols

To prepare the extracts, 20 g of dried *Ilex* leaves were transferred to a glass vial and 100 mL of pure methanol (Sigma-Aldrich, Steinheim, Germany) was added. Samples were shaken on a laboratory bench shaker for 24 h, then the primary extract was transferred to a round bottom flask, and extraction was repeated by adding another 100 mL of pure methanol. The samples were then centrifuged in a laboratory benchtop centrifuge (13,000 rpm, 10 min), and both extracts were filtered, combined, and concentrated in a laboratory evaporator. The extracts were diluted 1:200 and subjected to LC-MS analysis. The fraction prepared this way was then used for biological assays by getting the extract to the appropriate concentration in dedicated solvents (see Section 3.6 and Section 3.7 for information).

LC-MS/MS analysis was performed on a Shimadzu HPLC Prominence-i LC-2030C coupled to a triple quadrupole mass spectrometer (Shimadzu LCMS-8045). Separation of compounds in the extract was carried out on a reversed-phase column (Luna 3 µm C18 100A, 150 × 2.1 mm, Phenomenex, Torrance, Germany) at 40 °C. Two mobile phases were used for the analysis: water with 0.1% formic acid (eluent A) and acetonitrile (eluent B). The solvents used had a purity of >99.95%. The phase flow rate through the column was 0.35 mL/min, and the gradient was set as follows: start with 10% of solvent B, increase it to 20% over 5 min, then to 60% over 10 min, and then reduce it to 10% over 13 min and maintain it at 10% for 17 min. A 10 µL volume of the sample was used for the injection. The analysis was performed in triplicate. The analysis was carried out in negative electrospray ionisation (ESI) and the sputtering voltage was set at 4000 V. Bioactive compounds were analysed in the multiple reaction monitoring (MRM) mode; the MRM analysis parameters are given in Table 7. Other MS parameters were set as follows: nebulisation gas flow 3 L min^−1^, heating gas flow 10 L min^−1^, interface temperature 300 °C, desolvation temperature 526 °C, heat block temperature 400 °C, and drying gas flow 10 L min^−1^. Quantitative analysis was performed using pure standard substances and calibration curves. Interpretation of the results obtained was performed using the LabSolution Postrun Analysis version 5.12 software.

### 3.5. Saponins

Similarly, as in our previous UHPLC-HRMS and -MS/MS analyses, the saponin profiles in aqueous methanol extracts of leaf samples were compared using the described hardware settings [45], and some of the recorded compounds were identified as saponins according to the protocol already defined [46].

For analytical purposes, the samples were prepared as in the study by Włodarczyk and Gleńsk [46]. To obtain larger amounts of SAP fractions, powdered *I. aquifolium* Silver Queen leaves weighing 60 g were macerated twice for 24 h at room temperature using 600 mL of 70% methanol. After filtration, the extracts were combined and 30 g of lead (II) acetate trihydrate in 70% methanol was added to precipitate ballast substances. The extract was then centrifuged for 15 min at 3000 rpm (MPW). Residual lead ions in the saponin fraction were precipitated with dilute sulfuric acid. The resulting supernatant was neutralised and diluted with distilled water to a concentration of 40% methanol and centrifuged again. The supernatant was then applied, in two cycles, to a 20 g octadecyl SPE column. The saponin-rich fraction was eluted with pure methanol. The saponin fraction was concentrated to dryness using a vacuum evaporator at 40 °C and the glassy residue was then lyophilised. The fraction prepared in this way (SAP) was then used for biological assays by getting the extract to the appropriate concentration using dedicated solvents (see Section 3.6 and Section 3.7 for information).

A UHPLC Ultimate 3000 instrument (Thermo Fisher Scientific, Waltham, MA, USA) coupled to an ESI-qTOF Compact detector (Bruker Daltonics, Billerica, MA, USA) was used to analyse the saponin fraction. Saponin separation was achieved using a Kinetex C-18 column (150 × 2.1 mm, 2.6 μm; Phenomenex, Torrance, CA, USA). The flow rate through the column was set at 0.3 mL min^−1^. Water (eluent A) and acetonitrile (eluent B) slightly acidified with 0.1% formic acid were used for elution at 30 °C. The gradient was set as follows: 0–1 min (2–30% of B in A), 1–31 min (30–60% of B in A), 31–31.5 min (60–100% of B in A), and 31.5–35.5 min (60–100% of B in A). Later, the column was reconditioned. The detector was calibrated with sodium formate prior to each run. The detector was used in the negative mode. The general parameters of the instrument were set as follows: scanning range 50–2200 *m*/*z*, nebuliser pressure 1.5 bar, dry gas-nitrogen 7.0 L*min^−1^, temperature 200 °C, capillary voltage 2.2 kV, ion energy 5 eV, collision energy 10 eV vs. 30 eV, and low mass set 200 *m*/*z*. Analysis of the obtained mass spectra was carried out using Bruker Daltonics 4.1 data analysis software.

### 3.6. Antiviral Properties

Antiviral activity tests were conducted for the investigated extracts using enveloped human herpesvirus 1 (HSV-1—ATCC^®^ VR-1493™) and nonenveloped human adenovirus 5 (HAdV-5—ATCC^®^ VR-5™). Fractions of *I. aquifolium* Silver Queen were tested using EN 14476 [47]. This standard describes a quantitative suspension test for the evaluation of virucidal activity in the medical area (Phase 2/Step 1), mixing one part by volume (0.1 mL) of test virus suspension of HSV-1 (at a concentration of 10^12^ TCID_50_) or HAdV-5 virus (at a concentration of 10^8^ TCID_50_) with one part by volume of interfering substance (0.1 mL), and PBS (with bovine albumin), which simulates the non-sterile conditions under which the test product will potentially be used, and eight parts by volume of PLHA (polyphenol and phenolic acid extracts), SAP (saponin extract), or TERP (higher terpenoid extract) (concentration of 0.5% in distilled water for PLHA and SAP and 5% ethanol:water for TERP). Aliquots were taken at a specified contact time (60 min) and serial dilutions (up to 10^−12^) of each mixture were prepared. In eight repeats, 50 µL of each dilution was added to the microtiter plate containing a monolayer of confluent HeLa cells (ATCC^®^—CCL-2 ^TM^—human cervix carcinoma; ATCC, Manassas, VA, USA) or A549 cells (ATCC^®^—CCL-185^TM^—human lung epithelial carcinoma cells). The plate was observed daily for up to 4 days under an inverted microscope (Olympus Corp., Hamburg, Germany; Axio Observer, Carl Zeiss MicroImaging GmbH, Munich, Germany) for the development of viral cytopathic effect. Residual infectivity was then determined by comparing the titre (determined using TCID_50_) of the virus treated with the substance and the titre of the control virus. According to the standard, a substance has antiviral effectiveness if the titre is reduced within the recommended exposure time by ≥4 log10 steps (inactivation ≥ 99.99%).

In parallel, cytotoxicity tests were carried out as part of the procedure given in the standard and as a baseline for assessing the cytopathic effect caused by the virus. Since previous cytotoxicity tests using the SRB method identified the highest nontoxic concentrations, the virucidal assays used appropriate dilutions of fractions as starting points (concentration range 1–200 µg*mL^−1^). A number of dilutions, from 10^−1^ to 10^−12^, were prepared from each fraction, and 50 µL of each dilution was added to a microtiter plate containing a monolayer of confluent HeLa cells or A549 cells. The results were read in parallel with the virucidal results.

### 3.7. In Vitro Biological Activity

#### 3.7.1. In Vitro Cell Culture

The evaluation of biological activity was carried out on normal and tumour cell lines. Cells for the study were purchased from the European Collection of Authenticated Cell Cultures (ECACC) and stored in the cell culture laboratory biobank. Two weeks prior to the study, the cells were thawed and cultured under standard conditions of 37 °C, with 5% CO_2_ added to the incubator atmosphere. Normal mouse L929 cell lines and human fibroblasts (NHDF) were used in the study. Standard tumour cell lines, A549 (lung adenocarcinoma) and MCF7 (breast adenocarcinoma), and the colorectal tumour cell lines, LoVo and HT27, were used to study chemopreventive activity. Cells were cultured in standard DMEM (L929, NHDF), EMEM (A549 and MCF7), DMEM-F12 (LoVo), and McCoy’s 5A Medium (HT27) with serum and antibiotics. All cell culture reagents were purchased from Biological Industries (Beit-Haemek, Israel). Cell culture plastics and consumables from SPL Life Sciences (Gyeonggi-do, Republic of Korea) were used for the study.

#### 3.7.2. Assessment of Cell Growth and Cytotoxicity Using the SRB Test

In the experiment, cells were inoculated in 96-well plates at a density of 20,000 cells/well. After cell inoculation, plates were incubated at 37 °C, 5% CO_2_, 95% air, and 100% relative humidity for 24 h before adding test compounds. After 24 h, plates of each cell line were fixed with TCA to provide a measurement of the cell population for each cell line at the time of compound addition (Tz). Each *I. aquifolium* Silver Queen fraction (the highest content of biologically active compounds from the tested varieties) was diluted in DMSO to a concentration of 2%. The maximum solvent concentration did not exceed 0.5% and the solvent did not affect cell viability.

After adding the test compounds, the plates were incubated for 48 h in an incubator. After incubation, the assay was terminated by adding cold TCA. Cells were fixed by gently adding 50 μL of cold 50% (*w*/*v*) TCA (final concentration 10% TCA) and incubated for 60 min at 4 °C. The plates were washed five times with tap water and then air-dried. A 0.4% (*w*/*v*) solution of sulforhodamine B (SRB) (100 μL) in 1% acetic acid was added to each well and the plates incubated for 30 min at room temperature. After staining, the unbound dye was removed by washing five times with 1% acetic acid, and the plates were then dried. The bound dye was then dissolved in a 10 mM Trizma base and the absorbance read in a plate reader at 515 nm. Using absorbance measurements (zero time (Tz), control growth (C), and test growth in the presence of test compounds at different concentrations (Ti)), the percentage of cell growth was calculated. The percentage growth was calculated as ((Ti − Tz)/(C − Tz)) × 100. All reagents for cell growth assessment were purchased from Sigma-Aldrich, Germany.

### 3.8. Statistical Analysis

Statistical analyses were performed using Statistica software version 13.3 (StatSoft, Kraków, Poland) and PAST 4.17 by Ø. Hammer (Natural History Museum, University of Oslo). The results are expressed as means. Obtained data were subjected to analysis of variance (ANOVA) using a linear model procedure. Similarities and differences were determined using Tukey’s test. Principal component analysis (PCA) was performed based on the correlation matrix of the quantitative data obtained for whole samples.

## 4. Conclusions

The present study shows that the profiles of volatile compounds—triterpenoids, polyphenols, and phenolic acids—of samples of *I. paraguariensis* and varieties of *I. aquifolium* and *I. meserveae* are qualitatively similar. The dominant components in the volatile profiles (HS-SPME-Arrow) of the European cultivars were limonene, linalool, and *p*-cymene. For triterpenoids, they were ursolic acid and oleanolic acid. In the profiles of polyphenols and phenolic acids, the dominant components were dicaffeoylquinic acids as well as chlorogenic acid and its isomers. A metabolome of about 180 proposed saponins is presented for *I. aquifolium* and *I. meserveae*, enabling easy differentiation of samples. Fractions (terpenoids, saponins, polyphenols, and phenolic acids) of *I. aquifolium* Silver Queen, as the plant material with the highest content of bioactive compounds, were subjected to biological tests. Selected extracts showed strong biological activity, indicating potential in preventing and treating colon cancer. Additionally, the *I. aquifolium* extracts showed currently unpublished activity against HSV-1 and HAdV-5 viruses. In summary, European species of Ilex, i.e., *I. aquifolium* and *I. meserveae*, are promising sources of fractions of antiviral and antiproliferative effects but more comprehensive in vivo model studies are required.

## Figures and Tables

**Table 1:** Compositions of selected aroma compounds (HS-SPME-Arrow) from the leaves of *I. paraguariensis* and European cultivars of *I. aquifolium* and *I. meserveae*.

Compound	LRI Exp ^1^	LRI Lit ^2^	Ident. ^3^	*I. paraguariensis*	*I. aquifolium*			*I. meserveae*		
					Aurea Marginata	Silver Queen	Handsworth New Silver	Blue Prince	Blue Girl	Blue Princess
				Concentration (µg g^−1^) ^4^ d.w.						
Hexanal	800	801	LRI, MS, AS	0.65 ^5,a^	1.97 ^b^	2.06 ^b^	1.55 ^ab^	4.94 ^c^	5.17 ^c^	3.43 ^d^
Pentanoic acid + Isoamyl acetate	871	873	LRI, MS, AS	1.13 ^a^	0.77 ^a^	0.93 ^a^	0.95 ^a^	1.54 ^b^	1.69 ^b^	1.03 ^a^
α-Pinene	933	933	LRI, MS, AS	0.66 ^a^	2.27 ^b^	2.99 ^bd^	2.44 ^b^	4.75 ^c^	5.19 ^c^	4.20 ^c^
Sabinene	972	972	LRI, MS, AS	0.62 ^a^	1.21 ^ab^	2.07 ^bd^	1.55 ^ab^	3.17 ^cd^	3.76 ^c^	3.26 ^c^
β-Pinene	976	978	LRI, MS, AS	0.49 ^a^	1.58 ^ab^	2.55 ^bd^	1.72 ^ab^	4.09 ^c^	4.02 ^c^	3.58 ^c^
Hexanoic acid	979	980	LRI, MS, AS	2.01 ^a^	1.17 ^b^	1.62 ^ab^	1.25 ^ab^	3.30 ^c^	4.50 ^d^	1.95 ^ab^
6-methyl-Hept-5-en-2-one	985	986	LRI, MS, AS	0.49 ^a^	0.41 ^a^	0.61 ^ab^	0.41 ^a^	9.31 ^c^	1.14 ^c^	0.92 ^bc^
β-Myrcene	990	991	LRI, MS, AS	0.66 ^a^	1.64 ^ab^	2.51 ^bc^	1.84 ^ab^	3.68 ^cd^	4.49 ^d^	4.06 ^d^
(*E*,*E*)-2,4-Heptadienal	1012	1012	LRI, MS, AS	0.57 ^a^	1.78 ^ab^	2.82 ^b^	2.13 ^b^	4.47 ^c^	4.76 ^c^	4.68 ^c^
*p*-Cymene	1023	1025	LRI, MS, AS	5.54 ^a^	7.79 ^ab^	13.05 ^bc^	10.26 ^ab^	18.99 ^cd^	21.08 ^d^	21.42 ^d^
Limonene	1027	1030	LRI, MS, AS	7.04 ^a^	10.43 ^ab^	17.72 ^bc^	13.95 ^ab^	26.45 ^cd^	30.49 ^d^	31.45 ^d^
γ-Terpinene	1057	1058	LRI, MS, AS	0.28 ^a^	1.87 ^ab^	3.65 ^bc^	2.29 ^b^	5.49 ^cd^	5.61 ^d^	5.94 ^d^
Linalool	1098	1101	LRI, MS, AS	1.11 ^a^	8.73 ^b^	12.62 ^bc^	7.23 ^b^	18.26 ^cd^	19.87 ^d^	12.44 ^bc^
Terpinen-4-ol	1177	1184	LRI, MS, AS	0.32 ^a^	2.19 ^b^	3.22 ^b^	1.84 ^b^	4.76 ^c^	5.14 ^c^	3.05 ^b^
Linalyl acetate	1255	1250	LRI, MS, AS	0.34 ^a^	2.94 ^bc^	4.77 ^c^	2.36 ^ab^	7.43 ^d^	7.83 ^d^	4.43 ^bc^

^1^ Experimentally calculated linear retention index. ^2^ Literature-derived retention index according to Flavours and Fragrances of Natural and Synthetic Compounds 3.0 (FFNSC 3.0). ^3^ Method of identification: LRI—linear retention index, MS—mass spectrum, AS—authentic standard. ^4^ Calculated using peak area normalization against internal standard. ^5^ Values are mean; values followed by the same letter within a row are not significantly different (*p* > 0.05, Tukey’s test).

**Table 2:** Higher terpenoid and sterol contents in the leaves of *I. paraguariensis* and European cultivars of *I. aquifolium* and *I. meserveae*.

Compound ^1^	LRI Exp ^2^	LRI Lit ^3^	*I. paraguariensis*	*I. aquifolium*			*I. meserveae*		
				Aurea Marginata	Silver Queen	Handsworth New Silver	Blue Prince	Blue Girl	Blue Princess
			Concentration (mg g^−1^) ^4^ d.w.						
Stigmasterol	3280	3274	0.07 ^5,a^	0.03 ^d^	0.03 ^d^	0.04 ^c^	0.04 ^c^	0.06 ^b^	0.03 ^cd^
β-Sitosterol	3325	3346	0.01 ^a^	0.15 ^c^	0.08 ^b^	0.10 ^b^	0.08 ^b^	0.09 ^b^	0.11 ^b^
β-Amyrin	3338	3368	0.21 ^a^	1.00 ^d^	0.92 ^c^	0.71 ^b^	1.39 ^f^	1.60 ^g^	1.32 ^e^
α-Amyrin	3373	3406	0.40 ^a^	2.11 ^d^	2.60 ^e^	0.72 ^b^	1.37 ^c^	2.51 ^e^	2.57 ^e^
Lupeol	3384	3439	0.09 ^a^	0.48 ^d^	0.54 ^e^	0.20 ^b^	0.29 ^c^	0.80 ^f^	0.75 ^f^
Erythrodiol	3500	3501	0.11 ^a^	0.18 ^b^	0.16 ^b^	0.13 ^ab^	0.14 ^ab^	0.27 ^d^	0.21 ^c^
Uvaol	3535	3540	0.27 ^a^	0.34 ^d^	0.46 ^c^	0.19 ^f^	0.29 ^f^	0.67 ^e^	0.50 ^b^
Lup-20(29)-ene	3554	3558	0.01 ^a^	0.09 ^bc^	0.07 ^b^	0.07 ^bc^	0.01 ^a^	0.09 ^c^	0.09 ^c^
Oleanolic acid	3558	3562	1.54 ^a^	4.11 ^c^	4.11 ^c^	4.65 ^d^	4.08 ^c^	5.11 ^e^	3.14 ^b^
Betulinic acid	3583	3583	0.02 ^a^	0.28 ^e^	0.07 ^c^	0.26 ^e^	0.05 ^b^	1.80 ^d^	0.06 ^bc^
Ursolic acid	3611	3649	9.26 ^a^	11.27 ^c^	12.93 ^e^	12.06 ^d^	13.29 ^e^	10.70 ^b^	11.74 ^c^

^1^ Expressed as TMS derivatives. ^2^ Experimentally calculated linear retention index. ^3^ Literature-derived retention index of TMS derivatives, according to NIST23. ^4^ Calculated using peak area normalization against internal standard. ^5^ Values are mean; values followed by the same letter within a row are not significantly different (*p* > 0.05, Tukey’s test).

**Table 3:** Simple organic acids, phenolic acids, and polyphenols in the leaves of *I.paraguariensis* and European cultivars of *I. aquifolium* and *I. meserveae*.

Compound	*I. paraguariensis*	*I. aquifolium*			*I. meserveae*		
		Aurea Marginata	Silver Queen	Handsworth New Silver	Blue Prince	Blue Girl	Blue Princess
	Concentration (mg g^−1^) ^1^ d.w.						
Citric acid	2.17 ^a^	1.44 ^bc^	1.49 ^b^	1.30 ^bc^	1.28 ^c^	1.04 ^d^	1.30 ^bc^
Malic acid	1.26 ^a^	2.97 ^c^	2.03 ^ac^	2.03 ^ac^	0.77 ^b^	0.51 ^b^	1.93 ^a^
Quinic acid	1.76 ^a^	2.82 ^b^	4.94 ^e^	2.91 ^bc^	3.60 ^d^	1.81 ^a^	3.51 ^cd^
Caffeic acid	0.78 ^a^	0.99 ^b^	1.11 ^b^	1.16 ^b^	0.81 ^a^	0.72 ^a^	0.77 ^a^
Ferulic acid	0.98 ^a^	0.56 ^c^	0.66 ^c^	0.58 ^c^	0.28 ^d^	0.17 ^d^	0.84 ^b^
Chlorogenic acid (3-*O*-Caffeoylquinic acid)	13.48 ^a^	28.02 ^b^	26.75 ^b^	20.97 ^d^	17.53 ^c^	18.53 ^c^	38.96 ^e^
Cryptochlorogenic acid (4-*O*-Caffeoylquinic acid)	2.61 ^a^	4.69 ^c^	7.48 ^d^	3.64 ^b^	4.53 ^bc^	9.51 ^e^	3.81 ^bc^
Neochlorogenic acid (5-*O*-Caffeoylquinic acid)	7.47 ^a^	15.10 ^f^	12.36 ^e^	9.28 ^d^	8.37 ^c^	5.17 ^b^	21.62 ^g^
3,5-Di-*O*-caffeoylquinic acid	18.11 ^a^	3.90 ^d^	3.71 ^d^	5.97 ^c^	2.33 ^e^	1.32 ^f^	7.38 ^b^
4,5-Di-*O*-caffeoylquinic acid	6.11 ^a^	1.66 ^c^	1.79 ^c^	1.97 ^c^	0.94 ^d^	0.61 ^d^	3.37 ^b^
Rutin	4.10 ^2,a^	8.30 ^c^	11.80 ^f^	6.08 ^b^	5.10 ^ab^	6.05 ^b^	9.61 ^d^
Quercetin	1.51 ^a^	1.49 ^a^	1.28 ^b^	1.15 ^b^	1.12 ^b^	1.69 ^a^	1.59 ^a^

^1^ Quantification achieved in comparison with a pure standard and calibration curves. ^2^ Values are mean; values followed by the same letter within a row are not significantly different (*p* > 0.05, Tukey’s test).

**Table 4:** Comparison of antiviral properties of fractions of *I. aquifolium* Silver Queen leaves (expressed in logarithmic scale and % reduction).

Virus	PLHA	SAP	TERP
**HSV-1**	4 log (99.99%)	4 log (99.99%)	4 log (99.99%)
**HAdV-5**	4 log (99.99%)	4 log (99.99%)	2 log (99.00%)

PLHA—polyphenol and phenolic acid extract, SAP—saponin extract, TERP—higher terpenoids.

**Table 5:** Comparison of cytotoxic properties of fractions of *I. aquifolium* Silver Queen leaves on normal and cancer cell lines.

Cell Lines	L929	NHDF	A549	MCF7	LoVo	HT29
Extracts	GI50 ± SD value (µg·mL)					
**PLHA**	228.1 ± 36.7	239.7 ± 38.8	12.3 ± 3.0	12.3 ± 4.6	8.4 ± 1.3	34.4 ± 7.6
**SAP**	8.4 ± 0.9	360.2 ± 14.7	10.5 ± 3.8	7.1 ± 2.7	7.4 ± 1.1	16.1 ± 4.6
**TERP**	188.8 ± 7.4	183.5 ± 8.0	380.0 ± 6.9	124.2 ± 23.7	24.3 ± 3.9	795.2 ± 23.7

A549—human lung carcinoma cell line, HT29—human colorectal cancer cell line, L929—mouse subcutaneous connective tissue cell line (reference), LoVo—human colorectal cancer cell line, MCF7—human breast cancer cell line, NHDF—human fibroblasts line (reference); PLHA—polyphenols and phenolic acid extract, SAP—saponins extract, TERP—higher terpenoids.

**Table 6:** Dose ratio for growth inhibition of human-derived normal and tumour cell lines (therapeutic index).

Cell Lines	NHDF/A549	NHDF/MCF7	NHDF/LoVo	NHDF/HT29
Extracts	therapeutic index			
**PLHA**	19.5	19.5	28.5	7.0
**SAP**	34.3	50.7	48.7	22.4
**TERP**	0.5	1.5	7.6	0.2

**Table 7:** Multiple reaction monitoring (MRM) transitions and conditions of the mass spectrometer.

Compound	Precursor *m*/*z* (M − H)^−^	MRM Transitions *m*/*z* (Q_1_ → Q_3_)	Q_1_ Pre Bias (V)	Collision Energy	Q_3_ Pre Bias (V)
Citric acid	191.400	191.400 → 110.950	21.0	12.0	20.0
		191.400 → 86.950	13.0	17.0	17.0
		191.400 → 85.000	13.0	17.0	16.0
Malic acid	133.400	133.400 → 114.900	14.0	16.0	23.0
		133.400 → 71.000	14.0	15.0	12.0
Quinic acid	191.200	191.200 → 85.2500	14.0	21.0	30.0
		191.200 → 147.150	14.0	11.0	24.0
		191.200 → 93.100	15.0	23.0	29.0
Caffeic acid	301.200	301.200 → 151.000	22.0	21.0	15.0
		301.200 → 179.000	21.0	18.0	11.0
		301.200 → 121.000	22.0	27.0	24.0
Ferulic acid	193.400	193.400 → 134.000	12.0	14.0	26.0
		193.400 → 177.950	12.0	15.0	30.0
		193.400 → 149.050	12.0	13.0	14.0
Cryptochlorogenic acid (4-*O*-Caffeoylquinic acid)	353.000	353.000 → 191.300	17.0	15.0	19.0
		353.000 → 85.000	16.0	43.0	15.0
		353.000 → 93.050	17.0	46.0	17.0
Neochlorogenic acid (5-*O*-Caffeoylquinic acid)	353.000	353.000 → 191.300	17.0	15.0	19.0
		353.000 → 85.000	16.0	43.0	15.0
		353.000 → 93.050	17.0	46.0	17.0
Chlorogenic acid (3-*O*-Caffeoylquinic acid)	353.000	353.000 → 191.300	17.0	15.0	19.0
		353.000 → 85.000	16.0	43.0	15.0
		353.000 → 93.050	17.0	46.0	17.0
3,5-di-*O*-Caffeoylquinic acid	515.000	515.000 → 353.250	24.0	16.0	24.0
		515.000 → 191.300	24.0	31.0	18.0
		515.000 → 179.300	24.0	30.0	17.0
4,5-di-*O*-Caffeoylquinic acid	515.300	515.300 → 353.300	24.0	18.0	12.0
		515.300 → 179.100	24.0	30.0	17.0
		515.300 → 191.350	40.0	34.0	11.0
Rutin	609.300	609.300 → 300.150	30.0	39.0	19.0
		609.300 → 301.100	22.0	30.0	19.0
		609.300 → 271.250	22.0	55.0	17.0

## Data Availability

The original contributions presented in the study are included in the article/Supplementary Materials; further inquiries should be directed to the corresponding author.

## Supplementary Materials

The following supporting information can be downloaded at: https://www.mdpi.com/article/10.3390/molecules29133231/s1, Table S1: Aroma profiles (HS-SPME-Arrow) of various *Ilex* species; Table S2: List of triterpene saponins detected in the investigated *Ilex* species, their retention times, proposed formulae, fragmentations, and relative concentrations; Figure S1: Hierarchical clustering of *Ilex* taxons against their saponin constituents; Figure S2: Analysis of the effects of the tested extracts on the growth of L929 (A) and (B) NHDF cell lines using the SRB assay; Figure S3: Assessment of the cytotoxicity of the tested extracts against selected cancer lines: A549 (A), MCF7 (B), LoVo (C), and HT27 (D) using the SRB assay.
