# Supplementary material for: Antiviral and Cytotoxic Activities of Ilex aquifolium Silver Queen in the Context of Chemical Profiling of Two Ilex Species
Source: Molecules. 2024 Jul 8;29(13):3231. doi: 10.3390/molecules29133231 (PMC11243556; doi:10.3390/molecules29133231)
Supplement: Supplementary file 1 [file molecules-29-03231-s001.zip › Figures S2 i S3.pdf]

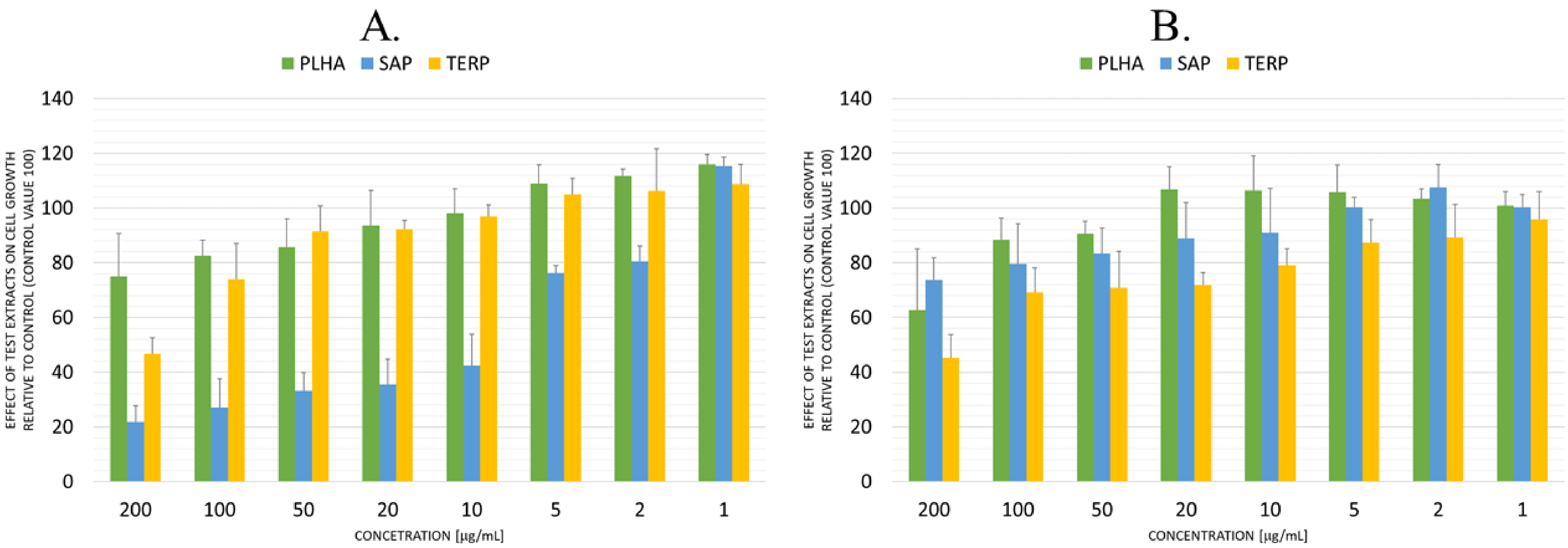

Figure S2. Effect on the growth of the L929 line (A.) and the NHDF line (B.) in the SRB assay.

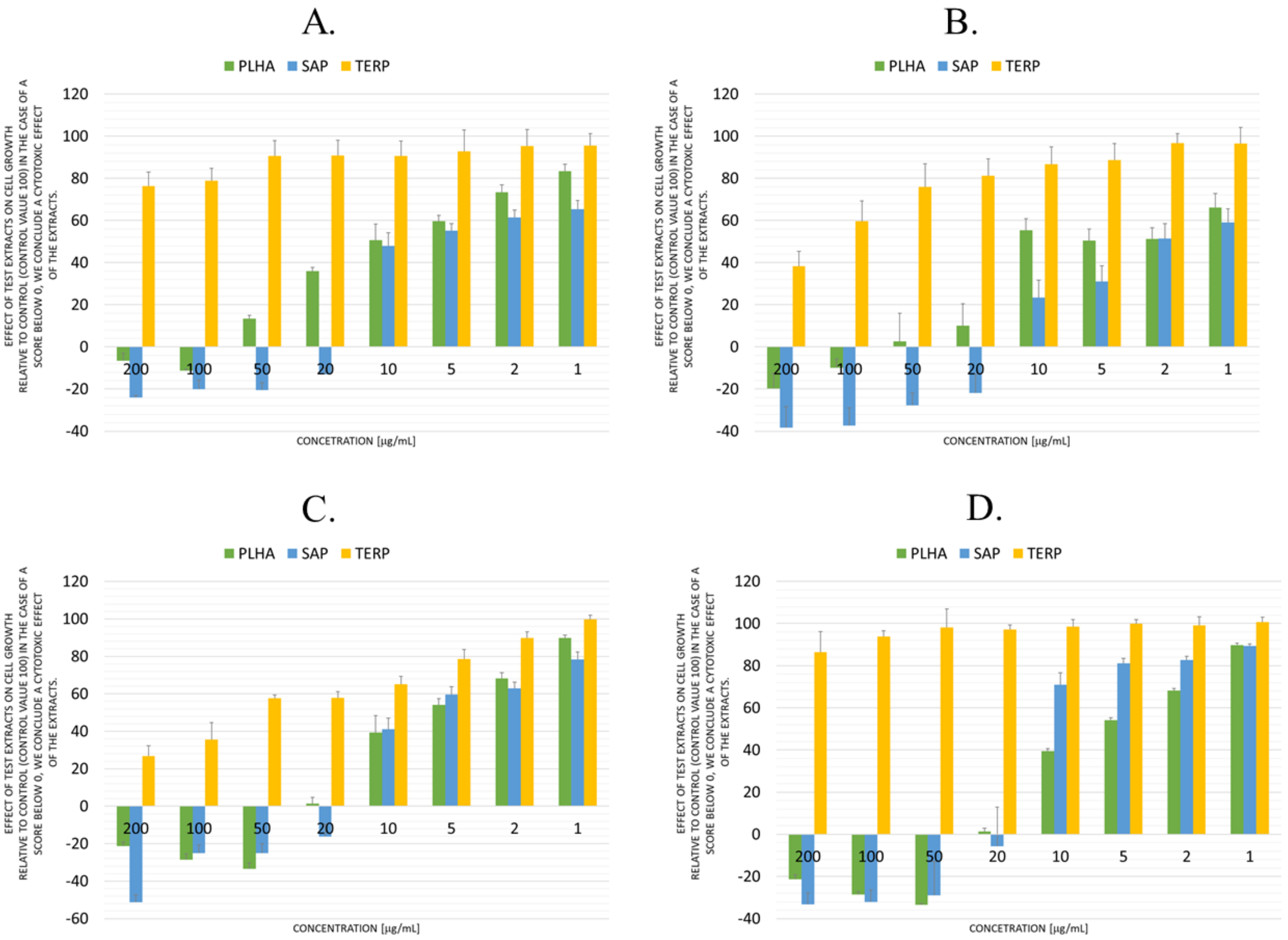

Figure S3. Assessment of the cytotoxicity of the tested extracts against selected cancer lines A549 (A.), MCF7 (B.), LoVo (C.) and HT27 (D.) in SRB assay. For results between 0 and 100, the figure shows the effect on cell growth inhibition. For values below 0, a cytotoxic effect is apparent by reducing the amount of cellular protein to the baseline amount when incubation with the test extracts is unleashed.
