# Supplementary material for: Antiviral and Cytotoxic Activities of Ilex aquifolium Silver Queen in the Context of Chemical Profiling of Two Ilex Species
Source: Molecules. 2024 Jul 8;29(13):3231. doi: 10.3390/molecules29133231 (PMC11243556; doi:10.3390/molecules29133231)
Supplement: Supplementary file 1 [file molecules-29-03231-s001.zip › Table S1.pdf]

	Compound	LRIexp	LRIlit	*I. paraguariensis*	*I. aquifolium* Aurea Marginata	*I. aquifolium* Silver Queen	*I. aquifolium* Handsworth New Silver	*I. meserveae* Blue Prince	*I. meserveae* Blue Girl	*I. meserveae* Blue Princess
				Concentration [µg*g^{-1}] d.w.						
1	Hexanal	800	831	0.65	1.97	2.06	1.55	4.94	5.17	3.43
2	2-Pentenal, 2-methyl-	828	842	0.31	0.11	0.12	0.09	0.28	0.27	0.09
3	(2*E*)-Hexenal	850	853	0.05	1.51	0.51	0.55	1.27	0.70	1.35
4	1-Hexanol	867	867	0.14	0.80	0.43	0.35	1.00	1.01	0.85
5	Pentanoic acid +Isoamyl acetate	871	904	1.13	0.77	0.93	0.95	1.54	1.69	1.03
6	2-Heptanone	898	898	0.19	0.09	0.09	0.10	0.18	0.24	0.15
7	Heptanal	902	906	0.24	0.10	0.12	0.09	0.19	0.24	0.15
8	α-Thujene	926	927	0.26	0.62	0.75	0.79	1.05	1.51	1.16
9	α-Pinene	933	933	0.66	2.27	2.99	2.44	4.75	5.19	4.20
10	2,2-Dimethyl-5-methylene norbornane	948	954	0.11	0.38	0.58	0.40	0.95	0.92	0.78
11	Benzaldehyde	959	960	0.24	0.44	0.43	0.45	0.76	0.90	0.61
12	1-Heptanol	967	970	0.11	0.01	0.02	0.02	0.03	0.03	0.03
13	Sabinene	972	972	0.62	1.21	2.07	1.55	3.17	3.76	3.26
14	β-Pinene	976	978	0.49	1.58	2.55	1.72	4.09	4.02	3.58
15	Hexanoic acid	979	980	2.01	1.17	1.62	1.25	3.30	4.50	1.95
16	6-methyl-Hept-5-en-2-one	985	986	0.49	0.41	0.61	0.41	0.93	1.14	0.92
17	Myrcene	990	991	0.66	1.64	2.51	1.84	3.68	4.49	4.06
18	Octanal	1002	1006	0.15	0.07	0.12	0.08	0.19	0.18	0.12
19	α-Phellandrene	1004	1007	0.20	0.78	1.23	0.92	1.68	2.19	2.20
20	(*E,E*)-2,4-Heptadienal	1012	1012	0.57	1.78	2.82	2.13	4.47	4.76	4.68
21	α-Terpinene	1016	1016	0.06	0.26	0.38	0.27	0.48	0.69	0.71
22	*p* -Cymene	1023	1025	5.54	7.79	13.05	10.26	18.99	21.08	21.42
23	Limonene	1027	1030	7.04	10.43	17.72	13.95	26.45	30.49	31.45
24	Eucalyptol	1032	1030	1.30	5.14	8.61	4.49	13.40	11.88	10.76
25	γ-vinyl-Valerolactone	1035	1039	0.11	0.01	0.01	0.01	0.04	0.03	0.01
26	(*E*)-β-Ocimene	1046	1046	0.08	0.46	0.81	0.41	1.11	1.21	1.05
27	γ-Terpinene	1057	1058	0.28	1.87	3.65	2.29	5.49	5.61	5.94
28	1-Octanol	1069	1076	0.11	0.09	0.08	0.09	0.12	0.22	0.12
29	cis-Linalool oxide	1069	1071	0.17	0.31	0.47	0.26	0.73	0.74	0.51
30	trans-Linalool oxide	1086	1084	0.11	0.22	0.34	0.19	0.53	0.57	0.37
31	Terpinolene	1087	1086	0.10	0.02	0.02	0.01	0.03	0.04	0.03
32	Linalool	1098	1101	1.11	8.73	12.62	7.23	18.26	19.87	12.44
33	Nonanal	1102	1107	0.23	0.38	0.59	0.44	0.93	0.88	0.70
34	1-Octen-3-ol, acetate	1109	1100	0.11	0.49	0.92	0.42	1.36	1.29	1.19
35	β-Thujone	1116	1118	0.03	0.05	0.07	0.05	0.10	0.09	0.07
36	Camphor	1144	1149	0.23	1.21	1.67	0.98	2.46	2.43	1.81
37	Menthone	1154	1158	0.60	0.41	0.70	0.36	1.02	0.98	0.81
38	Isomenthone	1164	1166	0.12	0.09	0.14	0.07	0.21	0.20	0.15
39	p-Menthan-1-ol	1173	1178	0.22	0.19	0.26	0.19	0.39	0.40	0.27
40	Terpinen-4-ol	1177	1184	0.32	2.19	3.22	1.84	4.76	5.14	3.05
41	*m* -Cymen-8-ol	1185	1183	0.05	0.14	0.18	0.14	0.30	0.31	0.15
42	α-Terpineol	1191	1195	0.15	0.93	1.20	0.89	1.78	2.12	1.06
43	methyl-Salicylate	1195	1192	0.29	1.08	1.97	0.99	3.02	3.13	2.00
44	Estragole	1199	1201	0.77	1.13	2.26	0.97	3.03	3.04	2.31
45	Decanal	1205	1208	0.04	0.07	0.10	0.09	0.16	0.17	0.12
46	Methylethylmaleimide	1232	1239	0.13	0.07	0.11	0.06	0.16	0.20	0.11
47	Thymol methyl ether	1235	1235	0.15	0.03	0.05	0.03	0.06	0.07	0.06
48	Cuminaldehyde	1241	1239	0.18	0.19	0.29	0.18	0.45	0.47	0.28
49	Carvone	1245	1246	0.13	0.41	0.61	0.39	0.93	0.96	0.60
50	Linalyl acetate	1255	1250	0.34	2.94	4.77	2.36	7.43	7.83	4.43
51	(2*E)* -Decenal	1261	1265	0.02	0.00	0.00	0.00	0.02	0.01	0.00
52	Geranial	1271	1268	0.04	0.19	0.30	0.18	0.47	0.58	0.26
53	Bornyl acetate	1286	1285	0.09	0.34	0.50	0.26	0.70	0.76	0.45
54	Carvacrol	1303	1300	0.33	0.22	0.25	0.32	0.48	0.48	0.24
55	α-Terpinyl acetate	1355	1349	0.37	0.19	0.27	0.17	0.42	0.45	0.24
56	Eugenol	1365	1357	0.09	0.11	0.12	0.12	0.23	0.25	0.07
57	Nerol acetate	1369	1364	0.60	0.03	0.04	0.02	0.06	0.06	0.04
58	cis-Geranyl acetate	1369	1361	0.32	0.06	0.05	0.04	0.08	0.08	0.05
59	α-Copaene	1383	1375	1.13	0.24	0.30	0.25	0.35	0.37	0.33
60	trans-Geranyl acetate	1386	1382	0.37	0.31	0.47	0.26	0.69	0.76	0.47
61	Tetradecane	1399	1400	0.32	0.12	0.15	0.11	0.23	0.21	0.14
62	2-Undecanone, 6,10-dimethyl-	1408	1405	0.75	0.00	0.00	0.00	0.00	0.00	0.00
63	methyl-Eugenol	1408	1403	0.06	0.01	0.02	0.01	0.02	0.03	0.01
64	α-cis-Bergamotene	1420	1421	0.03	0.03	0.04	0.02	0.06	0.09	0.03
65	Caryophyllene	1423	1419	0.14	0.25	0.35	0.21	0.47	0.50	0.29
66	α-Ionone	1434	1426	0.20	0.01	0.01	0.01	0.02	0.02	0.01
67	Isogermacrene D	1441	1447	0.01	0.03	0.03	0.02	0.05	0.05	0.03
68	α-Himachalene	1445	1551	0.06	0.07	0.11	0.06	0.15	0.15	0.09

69	trans-Geranylacetone	1457	1453	0.75	0.09	0.13	0.08	0.15	0.14	0.13
70	α-Humulene	1454	1464	0.32	0.02	0.02	0.01	0.03	0.02	0.02
71	Germacrene D	1486	1480	0.26	0.10	0.18	0.07	0.20	0.21	0.13
72	β-Ionon-5,6-epoxide	1489	1481	1.35	0.04	0.07	0.04	0.08	0.08	0.06
73	β-Selinene	1495	1492	0.29	0.01	0.01	0.01	0.02	0.02	0.01
74	α-Muurolene	1498	1499	0.03	0.02	0.02	0.02	0.04	0.06	0.02
75	β-Bisabolene	1515	1511	0.07	0.04	0.05	0.03	0.07	0.07	0.04
76	trans-Calamenene	1534	1529	0.08	0.01	0.01	0.01	0.07	0.01	0.06
77	2(4H)-Benzofuranone, 5,6,7,7a-tetrahydro-	1543	1538	2.02	0.19	0.26	0.17	0.35	0.34	0.25
78	Guaia-3,9-diene	1552	1556	0.26	0.01	0.01	0.01	0.02	0.02	0.01
79	trans-Dauca-4(11),7-diene	1556	1557	0.06	0.00	0.00	0.00	0.00	0.00	0.00
80	Caryophyllene oxide	1592	1587	0.07	0.04	0.05	0.03	0.08	0.07	0.04
81	Diethyl Phthalate	1596	1594	0.56	0.17	0.20	0.20	0.28	0.30	0.27
82	Guaiol	1599	1596	0.03	0.01	0.01	0.00	0.01	0.01	0.01
83	Humulene epoxide II	1602	1606	0.03	0.01	0.02	0.01	0.04	0.02	0.03
84	γ-Eudesmol	1639	1631	0.01	0.00	0.00	0.00	0.00	0.00	0.00
85	epi-γ-Eudesmol	1670	1662	0.54	0.07	0.07	0.08	0.14	0.15	0.11
86	2-Pentadecanone, 6,10,14-trimethyl-	1841	1844	0.41	0.02	0.02	0.02	0.06	0.05	0.02
