# Supplementary material for: Antiviral and Cytotoxic Activities of Ilex aquifolium Silver Queen in the Context of Chemical Profiling of Two Ilex Species
Source: Molecules. 2024 Jul 8;29(13):3231. doi: 10.3390/molecules29133231 (PMC11243556; doi:10.3390/molecules29133231)
Supplement: Supplementary file 1 [file molecules-29-03231-s001.zip › Table S2.pdf]

Table S2 UHPLC-ESI-HRMS and -MS/MS comparison of main leaf-saponin profiles of *Ilex aquifolium* cultivars, *Ilex × meserveae* cultivars and reference commercial *Ilex paraguariensis* (Mate).

No.	Compound name ([M]⁻, RT [min])	Cpd. neutral formula	MS/MS fragments [proposed pathway]	MS/MS aglycone	Measured [M]⁻ *m/z*	Err. [ppm]	Occurence frequency	Max RA %	Average RA % ± SD	SQ RA %	AM RA %	HNS RA %	BPR RA %	BP RA %	BG RA %	PA ref. RA %
1	1235.61, 6.05 min	C59H96O27	911 [M−(Hex+Hex)−H]−<br>765 [M−(Hex+Hex)−dHex−H]−<br>749 [M−(Hex+Hex)−Hex−H]−<br>603 [M−(Hex+Hex)−(dHex+Hex)−H]−<br>471 [M−(Hex+Hex)−(dHex+Hex)−Pen−H]−	471	1235.6103	-3.0	6	5.0	3.4 ± 0.9	4.1	3.9	5.0	3.0	3.2	1.4	—
2	927, 6.08 min	C47H76O18	—	—	927.4975	-1.8	2	3.1	2.2 ± 0.9	—	1.3	3.1	—	—	—	—
3	1089, 6.09 min	C53H86O23	—	—	1089.5521	-3.1	3	4.9	2.6 ± 1.5	2.2	0.7	4.9	—	—	—	—
4	927, 6.17 min	C47H76O18	—	—	927.4980	-2.2	3	3.1	2.3 ± 0.5	—	—	2.4	1.5	3.1	—	—
5	1089-S, 6.27 min	C50H90O23S	—	—	1089.5532	-1.0	1	3.8	3.8 ± 0.0	—	—	—	3.8	—	—	—
6	1131.56, 6.35 min	C55H88O24	—	—	1131.5625	-2.8	3	6.2	2.6 ± 2.4	0.5	1.0	6.2	—	—	—	—
7	927, 6.36 min	C47H76O18	—	—	927.4976	-1.8	4	9.9	6.9 ± 3.1	—	0.7	—	9.9	9.8	7.2	—
8	1089-S, 6.35 min	C50H90O23S	—	—	1089.5515	0.6	1	3.6	3.6 ± 0.0	—	—	—	—	—	3.6	—
9	781, 6.4 min	C41H66O14	—	—	781.4387	-0.9	3	6.1	4.7 ± 1.4	—	—	—	2.6	6.1	5.3	—
10	1235.61, 6.48 min	C59H96O27	—	—	1235.6108	1.4	1	1.7	1.7 ± 0.0	—	—	—	—	1.7	—	—
11	1001, 6.48 min	C48H74O22	—	—	1001.4616	-1.7	2	0.9	0.7 ± 0.1	—	0.6	0.9	—	—	—	—
12	927, 6.54 min	C47H76O18	—	—	927.4962	-0.3	2	1.5	1.0 ± 0.6	1.5	0.4	—	—	—	—	—
13	1131.56, 6.55 min	C55H88O24	—	—	1131.5624	-2.8	2	4.1	2.6 ± 1.5	1.1	—	4.1	—	—	—	—
14	1235.61, 6.62 min	C59H96O27	911 [M−(Hex+Hex)−H]−<br>765 [M−(Hex+Hex)−dHex−H]−<br>749 [M−(Hex+Hex)−Hex−H]−<br>603 [M−(Hex+Hex)−(dHex+Hex)−H]−<br>471 [M−(Hex+Hex)−(dHex+Hex)−Pen−H]−	471	1235.6109	-3.5	3	23.1	11.8 ± 7.5	—	11.5	23.1	0.9	—	—	—
15	1001, 6.67 min	C48H74O22	—	—	1001.4638	-3.9	6	13.7	5.0 ± 3.3	5.2	0.8	2.9	1.6	13.7	6.0	—
16	927, 6.68 min	C47H76O18	—	—	927.4994	-3.8	4	3.7	2.4 ± 0.8	2.5	0.7	3.7	2.7	—	—	—
17	1089, 6.72 min	C53H86O23	765 [M−(Hex+Hex)−H]−<br>603 [M−(Hex+Hex)−Hex−H]−<br>471 [M−(Hex+Hex)−Hex−Pen−H]−	471	1089.5514	-2.5	3	12.8	5.7 ± 4.7	—	1.4	12.8	3.0	—	—	—
18	781, 6.74 min	C41H66O14	—	—	781.4391	-1.4	3	2.3	2.0 ± 0.2	—	—	—	1.8	2.3	2.0	—
19	**1235.61, 6.79 min**	**C59H96O27**	911 [M−(Hex+Hex)−H]−<br>765 [M−(Hex+Hex)−dHex−H]−<br>749 [M−(Hex+Hex)−Hex−H]−<br>603 [M−(Hex+Hex)−(dHex+Hex)−H]−<br>471 [M−(Hex+Hex)−(dHex+Hex)−Pen−H]−	471	1235.6116	-4.0	**6**	**100.0**	**40.5 ± 28.7**	13.6	**32.2**	**67.0**	24.6	**100.0**	5.6	—
20	839, 6.85 min	C42H64O17	—	—	839.4082	-1.4	6	3.8	2.0 ± 0.7	3.8	1.3	1.8	1.8	2.1	0.9	—
21	1089, 6.88 min	C53H86O23	825 [M−264−H]−<br>663 [M−264−Hex−H]−<br>487 [M−264−Hex−HexA−H]−	487	1089.5516	-2.6	6	10.2	5.6 ± 2.8	2.8	2.5	10.2	6.6	8.5	3.2	—
22	927, 6.9 min	C47H76O18	—	—	927.4998	-4.2	5	6.7	3.7 ± 1.3	—	2.0	3.4	4.0	6.7	2.6	—
23	825, 6.9 min	C42H66O16	—	—	825.4294	-1.9	3	3.4	2.3 ± 0.7	2.2	1.3	3.4	—	—	—	—
24	779, 6.91 min	C41H64O14	—	—	779.4227	-0.4	3	2.1	1.5 ± 0.4	1.4	0.8	2.1	—	—	—	—
25	825, 7.01 min	C42H66O16	—	—	825.4289	-1.3	3	3.3	1.9 ± 1.0	3.3	1.1	1.3	—	—	—	—
26	839, 7.03 min	C42H64O17	663 [M−HexA−H]−<br>501 [M−HexA−Hex−H]−	501	839.4088	-2.1	7	14.8	4.3 ± 3	4.3	0.5	0.4	3.2	14.8	3.7	3.1
27	1089, 7.07 min	C53H86O23	—	—	1089.5516	-2.7	6	6.7	3.7 ± 2.4	1.2	0.5	2.3	5.3	6.3	6.7	—
28	927, 7.07 min	C47H76O18	765 [M−Hex−H]−<br>619 [M−Hex−dHex−H]−<br>487 [M−Hex−dHex−Pen−H]−	487	927.4987	-3.0	6	15.9	8.1 ± 5.2	4.0	0.9	3.6	11.6	12.4	15.9	—
29	**1131.56, 7.08 min**	**C55H88O24**	—	—	1131.5629	-3.2	**2**	**30.9**	17.7 ± 13.2	—	4.4	**30.9**	—	—	—	—
30	1073, 7.09 min	C53H86O22	—	—	1073.5577	-3.6	3	7.4	4.4 ± 2.0	—	—	—	7.4	4.2	1.6	—
31	839, 7.12 min	C42H64O17	663 [M−HexA−H]−<br>501 [M−HexA−Hex−H]−	501	839.4090	-2.3	7	8.7	4.0 ± 1.9	2.4	1.5	5.7	2.2	8.7	3.7	3.5
32	1235.61, 7.13 min	C59H96O27	—	—	1235.6111	-3.6	2	2.7	2.2 ± 0.5	—	—	—	2.7	—	1.7	—
33	781, 7.13 min	C41H66O14	—	—	781.4394	-1.8	6	11.7	6.7 ± 2.8	11.7	1.9	9.8	4.0	7.1	5.9	—
34	825, 7.15 min	C42H66O16	—	—	825.4292	-1.7	1	3.1	3.1 ± 0.0	—	—	3.1	—	—	—	—
35	927, 7.18 min	C47H76O18	—	—	927.4993	-3.7	7	19.6	6.9 ± 3.7	3.9	2.3	19.6	6.0	6.7	7.1	2.7
36	1089, 7.19 min	C53H86O23	—	—	1089.5511	-2.2	6	9.0	3.5 ± 1.9	1.1	1.1	9.0	2.8	3.6	3.3	—
37	823, 7.24 min	C43H68O15	—	—	823.4493	-0.9	2	4.7	2.7 ± 2.0	—	0.7	4.7	—	—	—	—
38	781, 7.24 min	C41H66O14	—	—	781.4383	-0.4	5	10.0	3.2 ± 2.7	—	0.9	10.0	1.5	2.1	1.5	—
39	971, 7.29 min	C48H76O20	647 [M−(Hex+Hex)−H]−<br>471 [M−(Hex+Hex)−HA−H]−	471	971.4887	-3.1	3	6.5	3.6 ± 2.0	—	—	—	6.5	2.3	1.9	—
40	**1131.56, 7.31 min**	**C55H88O24**	765 [M−(Hex+Hex+Ac)−H]−<br>747 [M−(Hex+Hex+Ac)−H2O−H]−<br>603 [M−(Hex+Hex+Ac)−Hex−H]−<br>471 [M−(Hex+Hex+Ac)−Hex−Pen−H]−	471	1131.5629	-3.2	**3**	**39.4**	15.4 ± 16.0	0.7	6.1	**39.4**	—	—	—	—
41	1235.61, 7.3 min	C59H96O27	—	—	1235.6119	-3.5	1	2.1	2.1 ± 0.0	—	—	—	—	2.1	—	—
42	1073, 7.41 min	C53H86O22	749 [M−(Hex+Hex)−H]−<br>705 [M−(Hex+Hex)−CO2−H]−<br>501 [M−(Hex+Hex)−CO2−(Hex+Ac)−H]−<br>453 [M−(Hex+Hex)−CO2−(Hex+Ac)−HCOOH−H]−	453	1073.5563	-2.3	4	18.6	9.1 ± 4.9	—	—	7.1	18.6	9.4	1.2	—
43	823, 7.43 min	C43H68O15	781 [M−42−H]−<br>619 [M−42−Hex−H]−<br>487 [M−42−Hex−Pen−H]−	487	823.4490	-0.6	3	6.7	2.7 ± 2.7	6.7	0.6	0.8	—	—	—	—
44	**1235.61, 7.51 min**	**C59H96O27**	911 [M−(Hex+Hex)−H]−<br>765 [M−(Hex+Hex)−dHex−H]−<br>749 [M−(Hex+Hex)−Hex−H]−<br>603 [M−(Hex+Hex)−(dHex+Hex)−H]−<br>471 [M−(Hex+Hex)−(dHex+Hex)−Pen−H]−	471	1235.6104	-3.0	**4**	**28.1**	10.1 ± 9.0	1.3	—	—	**28.1**	4.1	6.9	—
45	971, 7.48 min	C48H76O20	—	—	971.4880	-2.4	6	7.2	4.1 ± 1.8	4.2	1.8	2.6	2.5	6.2	7.2	—
46	**1131.56, 7.54 min**	**C55H88O24**	765 [M−(Hex+Hex+Ac)−H]−<br>747 [M−(Hex+Hex+Ac)−H2O−H]−<br>603 [M−(Hex+Hex+Ac)−Hex−H]−<br>471 [M−(Hex+Hex+Ac)−Hex−Pen−H]−	471	1131.5626	-2.9	**3**	**26.4**	10.2 ± 10.9	2.1	2.0	**26.4**	—	—	—	—
47	823, 7.54 min	C43H68O15	781 [M−42−H]−<br>619 [M−42−Hex−H]−<br>487 [M−42−Hex−Pen−H]−	487	823.4499	-1.6	3	9.6	4.6 ± 3.3	3.2	1.1	9.6	—	—	—	—
48	1089, 7.64 min	C53H86O23	—	—	1089.5516	-2.7	1	4.2	4.2 ± 0.0	—	—	—	—	—	4.2	—
49	927, 7.68 min	C47H76O18	—	—	927.4990	-3.4	2	5.7	4.4 ± 1.3	3.1	—	5.7	—	—	—	—
50	**825, 7.71 min**	**C42H66O16**	663 [M−Hex−H]−<br>619 [M−Hex−CO2−H]−<br>487 [M−Hex−HexA−H]−	487	825.4277	0.1	**7**	**88.7**	19.5 ± 20.2	6.5	2.1	1.5	**88.7**	21.1	7.6	9.4
51	1073, 7.78 min	C53H86O22	911 [M−Hex−H]−<br>765 [M−dHex−H]−<br>749 [M−Hex−H]−<br>603 [M−(Hex+dHex)−H]−<br>471 [M−(Hex+dHex)−Pen−H]−	471	1073.5567	-2.7	3	6.2	5.4 ± 0.6	—	6.2	5.5	4.4	—	—	—
52	1235.61, 7.9 min	C59H96O27	—	—	1235.6098	-2.6	1	2.3	2.3 ± 0.0	—	—	—	2.3	—	—	—
53	**927, 7.92 min**	**C47H76O18**	765 [M−Hex−H]−<br>603 [M−Hex−Hex−H]−<br>471 [M−Hex−Hex−Pen−H]−	471	927.4971	-1.3	**6**	**68.4**	18.4 ± 16.7	4.5	10.8	**68.4**	7.0	11.5	8.0	—
54	969, 7.98 min	C49H78O19	—	—	969.5092	-2.8	1	1.5	1.5 ± 0.0	1.5	—	—	—	—	—	—
55	887-S, 7.96 min	C43H68O17S	725 [M−Hex−H]−	—	879.4133	-3.2	3	3.2	1.9 ± 1.2	3.2	2.4	0.2	—	—	—	—
56	879, 7.98 min	C46H72O16	—	—	879.4753	-0.6	2	1.5	0.9 ± 0.5	1.5	0.4	—	—	—	—	—
57	1131.56, 8.05 min	C55H88O24	—	—	1131.5614	-1.8	2	8.7	5.2 ± 3.4	1.8	—	8.7	—	—	—	—
58	**1073, 8.11 min**	**C53H86O22**	911 [M−Hex−H]−<br>765 [M−dHex−H]−<br>749 [M−Hex−H]−<br>603 [M−(Hex+dHex)−H]−<br>471 [M−(Hex+dHex)−Pen−H]−	471	1073.5584	-4.3	**7**	**85.7**	**58.3 ± 22.4**	**64.8**	**29.5**	**53.0**	**85.7**	**75.8**	**85.3**	14.0
59	**809, 8.11 min**	**C42H66O15**	—	—	809.4354	-3.1	**6**	**36.5**	18.7 ± 9.7	15.2	3.4	8.6	**26.5**	**36.5**	22.2	—
60	927, 8.13 min	C47H76O18	—	—	927.4946	1.4	1	5.0	5.0 ± 0.0	—	—	—	—	—	—	5.0
61	765, 8.25 min	C41H66O13	603 [M−Hex−H]−	—	765.4419	1.6	1	9.1	9.1 ± 0.0	—	—	—	—	—	—	9.1
62	**927, 8.29 min**	**C47H76O18**	765 [M−Hex−H]−<br>603 [M−Hex−Hex−H]−<br>471 [M−Hex−Hex−Pen−H]−	471	927.4964	-0.6	**7**	**100.0**	**64.3 ± 28.7**	**100.0**	**26.5**	**100.0**	**77.6**	**80.2**	**59.8**	6.4
63	1131.56, 8.35 min	C55H88O24	—	—	1131.5576	1.5	1	1.7	1.7 ± 0.0	—	—	—	—	—	—	1.7
64	1101, 8.5 min	C54H86O23	939 [M−Hex−H]−	—	1101.5492	-0.4	1	2.0	2.0 ± 0.0	—	—	—	—	—	—	2.0
65	1381, 8.54 min	C65H106O31	895 [M−(Hex+Hex+Hex)−H]−<br>733 [M−(Hex+Hex+Hex)−Hex−H]−<br>587 [M−(Hex+Hex+Hex)−Hex−dHex−H]−<br>455 [M−(Hex+Hex+Hex)−Hex−dHex−Pen−H]−	455	1381.6658	-0.9	1	13.6	13.6 ± 0.0	—	—	—	—	—	—	13.6
66	927, 8.54 min	C47H76O18	765 [M−Hex−H]−<br>603 [M−Hex−Hex−H]−	—	927.4974	-1.7	5	9.6	6.0 ± 2.2	5.0	—	9.6	7.9	2.3	5.4	—
67	781, 8.58 min	C41H66O14	—	—	781.4398	-2.4	5	3.9	1.8 ± 1.4	3.1	0.8	3.9	—	0.8	0.3	—
68	**1397, 8.63 min**	**C61H106O35**	1073 [M−(Hex+Hex)−H]−<br>911 [M−(Hex+Hex)−Hex−H]−<br>749 [M−(Hex+Hex)−Hex−Hex−H]−<br>603 [M−(Hex+Hex)−Hex−Hex−dHex−H]−<br>471 [M−(Hex+Hex)−Hex−Hex−dHex−Pen−H]−	471	1397.6468	-1.9	**4**	**39.5**	12.7 ± 13.4	0.9	1.4	9.2	—	**39.5**	—	—
69	969, 8.63 min	C49H78O19	927 [M−Ac−H]−<br>807 [M−Hex−H]−<br>765 [M−Ac−Hex−H]−<br>603 [M−Ac−Hex−Hex−H]−<br>471 [M−Ac−Hex−Hex−Pen−H]−	471	969.5087	-2.3	2	23.0	14.1 ± 8.9	—	5.2	23.0	—	—	—	—
70	1073, 8.7 min	C53H86O22	—	—	1073.5537	0.1	2	4.7	2.7 ± 2.0	—	—	4.7	—	—	0.7	—
71	663, 8.72 min	C36H56O11	—	—	663.3769	-2.9	3	3.3	2.8 ± 0.5	—	—	—	2.1	3.3	3.2	—
72	1131.56, 8.74 min	C55H88O24	—	—	1131.5603	-0.9	2	6.2	3.9 ± 2.3	1.7	—	—	—	—	—	6.2
73	969, 8.74 min	C49H78O19	927 [M−Ac−H]−<br>765 [M−Ac−Hex−H]−<br>603 [M−Ac−Hex−Hex−H]−<br>471 [M−Ac−Hex−Hex−Pen−H]−	471	969.5109	-4.6	2	11.0	7.1 ± 3.9	3.1	—	—	—	—	—	11.0
74	781, 8.79 min	C41H66O14	—	—	781.4396	-2.0	4	3.9	2.2 ± 0.9	1.1	—	2.0	—	1.7	3.9	—
75	1101, 8.88 min	C54H86O23	939 [M−Hex−H]−<br>897 [M−(Hex+Ac)−H]−<br>765 [M−(Hex+Ac)−Pen−H]−<br>603 [M−(Hex+Ac)−Pen−Hex−H]−<br>471 [M−(Hex+Ac)−Pen−Hex−Pen−H]−	471	1101.5487	0.0	1	5.2	5.2 ± 0.0	—	—	—	—	—	—	5.2
76	1219.61, 8.89 min	C59H96O26	895 (100) [M−Hex−Hex−H]−	—	1219.6147	-2.4	2	0.6	0.5 ± 0.0	—	0.5	0.6	—	—	—	—
77	663, 8.95 min	C36H56O11	501 [M−Hex−H]−	501	663.3762	-1.9	6	14.4	4.9 ± 3.2	—	2.4	14.4	1.6	3.8	3.5	3.8
78	**969, 9.07 min**	**C49H78O19**	927 [M−Ac−H]−<br>807 [M−Hex−H]−<br>765 [M−Ac−Hex−H]−<br>603 [M−Ac−Hex−Hex−H]−<br>471 [M−Ac−Hex−Hex−Pen−H]−	471	969.5101	-3.8	**7**	**44.9**	12.4 ± 11.9	20.6	13.2	**44.9**	2.9	2.9	1.3	1.1
79	**809, 9.07 min**	**C42H66O15**	647 [M−Hex−H]−<br>471 [M−Hex−HexA−H]−	471	809.4334	-0.7	**1**	**50.4**	**50.4 ± 0.0**	—	—	—	—	—	—	**50.4**
80	**1219.61, 9.16 min**	**C59H96O26**	895 (100) [M−Hex−Hex−H]−<br>733 [M−Hex−Hex−Hex−H]−<br>587 [M−Hex−Hex−Hex−dHex−H]−<br>455 [M−Hex−Hex−Hex−dHex−Pen−H]−	455	1219.6157	-3.3	**7**	**100.0**	**45.5 ± 31.9**	20.2	10.3	21.1	**100.0**	**91.8**	18.9	**56.3**
81	1073, 9.22 min	C53H86O22	—	—	1073.5564	-2.4	3	12.3	5.1 ± 4.8	—	—	—	12.3	1.4	1.6	—
82	**911, 9.24 min**	**C47H76O17**	—	—	911.5038	-3.1	**7**	**37.7**	16.9 ± 8.5	14.2	3.3	23.4	37.7	19.4	16.3	3.8
83	**663, 9.24 min**	**C36H56O11**	501 [M−Hex−H]−	501	663.3742	1.2	**5**	**31.5**	13.6 ± 9.1	4.8	—	—	2.8	18.4	10.7	31.5
84	809, 9.27 min	C42H66O15	647 [M−Hex−H]−<br>471 [M−Hex−HexA−H]−	471	809.4338	-1.0	1	22.8	22.8 ± 0.0	—	—	—	—	—	—	22.8
85	663, 9.35 min	C36H56O11	—	—	663.3759	-1.4	2	10.2	5.4 ± 4.8	—	0.6	10.2	—	—	—	—
86	1073, 9.34 min	C53H86O22	—	—	1073.5555	-1.5	1	3.3	3.3 ± 0.0	—	—	3.3	—	—	—	—
87	**1073, 9.52 min**	**C53H86O22**	911 [M−Hex−H]−<br>765 [M−dHex−H]−<br>749 [M−Hex−H]−<br>603 [M−(Hex+dHex)−H]−<br>471 [M−(Hex+dHex)−Pen−H]−	471	1073.5566	-2.6	**7**	**40.7**	15.2 ± 7.5	12.6	3.4	**40.7**	10.4	15.6	15.6	8.4
88	**969, 9.5 min**	**C49H78O19**	927 [M−Ac−H]−<br>807 [M−Hex−H]−<br>765 [M−Ac−Hex−H]−<br>603 [M−Ac−Hex−Hex−H]−<br>471 [M−Ac−Hex−Hex−Pen−H]−	471	969.5112	-4.9	**7**	**67.0**	17.9 ± 19.9	**38.5**	10.6	**67.0**	2.4	2.8	0.4	3.9
89	927, 9.5 min	C47H76O18	—	—	927.4954	0.6	1	4.7	4.7 ± 0.0	—	—	—	—	—	—	4.7
90	765, 9.5 min	C41H66O13	603 [M−Hex−H]−<br>471 [M−Hex−Pen−H]−	471	765.4437	-0.9	1	17.0	17 ± 0.0	—	—	—	—	—	—	17.0

→ continued

No.	Compound name ([M]⁻, RT [min])	Cpd. neutral formula	MS/MS fragments [proposed pathway]	MS/MS aglycone	Measured [M]⁻ *m/z*	Err. [ppm]	Occurence frequency	Max RA %	Average RA % ± SD	SQ RA %	AM RA %	HNS RA %	BPR RA %	BP RA %	BG RA %	PA ref. RA %
91	1219.61, 9.97 min	C59H96O26	—	—	1219.6159	-3.4	1	1.7	1.7 ± 0.0	—	—	—	—	—	1.7	—
92	969, 9.98 min	C49H78O19	645 [M−(Hex+Hex)−H]−	—	969.5044	2.2	6	23.7	6.0 ± 6.2	7.1	0.2	23.7	2.1	1.2	1.9	—
93	1409, 9.85 min	C66H106O32	complicated	455	1409.6628	-2.4	1	3.5	3.5 ± 0.0	—	—	—	—	—	—	3.5
94	1235.58, 10.01 min	C62H92O25	—	—	1235.5904	-4.0	5	1.6	0.9 ± 0.5	1.1	0.3	—	1.6	1.1	0.2	—
95	879, 10.1 min	C46H72O16	—	—	879.4778	-3.5	2	1.7	0.9 ± 0.8	1.7	0.1	—	—	—	—	—
96	887-S, 10.13 min	C43H68O17S	725 [M−Hex−H]−	—	879.4120	-1.8	3	18.2	6.8 ± 7.6	—	18.2	1.3	0.9	—	—	—
97	1277, 10.21 min	C61H98O28	complicated	455	1277.6183	-0.8	1	4.6	4.6 ± 0.0	—	—	—	—	—	—	4.6
98	779, 10.24 min	C41H64O14	—	—	779.4243	-2.5	3	6.0	3.1 ± 1.9	2.2	1.1	6.0	—	—	—	—
99	809, 10.27 min	C42H66O15	647 [M−Hex−H]−<br>471 [M−Hex−HexA−H]−	471	809.4348	-2.4	4	6.0	2.6 ± 1.7	2.2	—	—	6.0	1.2	1.1	—
100	851, 10.36 min	C44H68O16	791 [M−AA−H]−<br>629 [M−AA−Hex−H]−<br>585 [M−AA−Hex−CO2−H]−<br>453 [M−AA−Hex−HexA/(CO2+Pen)−H]−	453	851.4449	-1.6	6	5.3	2.4 ± 1.0	2.5	1.6	5.3	1.6	2.0	1.3	—
101	1247, 10.47 min	C60H96O27	complicated	455	1247.6079	-1.0	1	12.6	12.6 ± 0.0	—	—	—	—	—	—	12.6
102	**1115, 10.48 min**	**C55H88O23**	749 [M−(Hex+Hex+Ac)−H]−<br>731 [M−(Hex+Hex+Ac)−H2O−H]−<br>587 [M−(Hex+Hex+Ac)−Hex−H]−<br>455 [M−(Hex+Hex+Ac)−Hex−Pen−H]−	455	1115.5678	-3.1	**3**	**32.8**	12.6 ± 13.4	2.3	2.9	**32.8**	—	—	—	—
103	1235.58, 10.56 min	C62H92O25	—	—	1235.5897	-3.4	7	6.0	2.4 ± 1.8	1.3	0.5	1.2	5.2	1.8	0.7	6.0
104	851, 10.66 min	C44H68O16	—	—	851.4443	-1.0	6	1.3	0.9 ± 0.3	1.1	0.3	0.9	1.1	1.3	0.8	—
105	1235.58, 10.7 min	C62H92O25	—	—	1235.5917	-5.1	7	5.3	1.9 ± 1.2	1.4	0.3	2.3	2.1	1.3	0.3	5.3
106	**887-S, 10.71 min**	**C43H68O17S**	725 [M−Hex−H]−	—	879.4126	-2.4	**6**	**100.0**	**38.9 ± 32.2**	**74.4**	**100.0**	14.5	11.9	**30.6**	1.8	—
107	839, 10.74 min	C42H64O17	795 [M−CO2−H]−<br>501 [M−CO2−dHex−dHex−H]−	501	839.4088	-2.0	2	9.8	8.5 ± 1.3	—	—	—	—	7.2	9.8	—
108	927, 10.9 min	C47H76O18	781 [M−dHex−H]−<br>765 [M−Hex−H]−<br>619 [M−(Hex+dHex)−H]−<br>487 [M−(Hex+dHex)−Pen−H]−	487	927.4993	-3.6	3	23.7	13.9 ± 6.6	—	—	—	7.2	10.8	23.7	—
109	**1057, 10.89 min**	**C53H86O21**	733 [M−Hex−Hex−H]−<br>587 [M−Hex−Hex−dHex−H]−<br>455[M−Hex−Hex−dHex−Pen−H]−	455	1057.5602	-1.2	**7**	**83.7**	**36.1 ± 21.9**	12.4	1.7	17.6	**37.9**	**83.7**	**50.3**	**49.3**
110	927, 11.04 min	C47H76O18	—	—	927.4988	-3.2	3	9.7	5.0 ± 3.1	—	—	—	2.5	2.8	9.7	—
111	**1235.58, 11.07 min**	**C62H92O25**	1073 [M−Hex−H]−<br>911 [M−Hex−Hex−H]−<br>765 [M−Hex−Hex−dHex−H]−<br>749 [M−Hex−Hex−Hex−H]−<br>603 [M−Hex−Hex−(Hex+dHex)−H]−<br>471 [M−Hex−Hex−(Hex+dHex)−Pen−H]−	471	1235.5889	-2.8	**7**	**40.9**	21.9 ± 10.1	**31.1**	7.3	**29.2**	18.0	**40.9**	7.8	19.0
112	1089.53, 11.14 min	C56H82O21	927 [M−Hex−H]−<br>765 [M−Hex−Hex−H]−<br>603 [M−Hex−Hex−Hex−H]−<br>471 [M−Hex−Hex−Hex−Pen−H]−	471	1089.5298	-2.1	4	16.0	5.1 ± 5.4	2.0	0.8	16.0	—	1.8	—	—
113	**1115, 11.17 min**	**C55H88O23**	749 [M−(Hex+Hex+Ac)−H]−<br>731 [M−(Hex+Hex+Ac)−H2O−H]−<br>587 [M−(Hex+Hex+Ac)−Hex−H]−<br>455 [M−(Hex+Hex+Ac)−Hex−Pen−H]−	455	1115.5666	-2.0	**4**	**25.2**	9.5 ± 7.8	6.6	1.1	**25.2**	—	—	—	5.2
114	1033-S, 11.27 min	C49H78O21S	709 [M−(Hex+Hex)−H]−<br>649 [M−(Hex+Hex)−AA−H]−<br>569 [M−(Hex+Hex)−AA−SO3−H]−<br>437 [M−(Hex+Hex)−AA−SO3−Pen−H]−	437	1033.4696	-1.2	1	0.8	0.8 ± 0.0	—	0.8	—	—	—	—	—
115	**1115, 11.32 min**	**C55H88O23**	749 [M−(Hex+Hex+Ac)−H]−<br>731 [M−(Hex+Hex+Ac)−H2O−H]−<br>587 [M−(Hex+Hex+Ac)−Hex−H]−<br>455 [M−(Hex+Hex+Ac)−Hex−Pen−H]−	455	1115.5655	-1.1	**1**	**36.5**	**36.5 ± 0.0**	—	—	—	—	—	—	**36.5**
116	645, 11.48 min	C37H58O9	—	—	645.4032	-3.7	3	1.6	1.1 ± 0.6	1.5	0.1	1.6	—	—	—	—
117	**777, 11.5 min**	**C41H62O14**	615 [M−Hex−H]−<br>439 [M−Hex−HexA−H]−	439	777.4073	-0.8	**1**	**39.4**	**39.4 ± 0.0**	—	—	—	—	—	—	**39.4**
118	663, 11.53 min	C36H56O11	—	—	663.3757	-1.1	3	4.5	3.3 ± 1.3	—	—	—	1.4	4.5	4.0	—
119	1235.58, 11.56 min	C62H92O25	—	—	1235.5862	-0.6	2	0.8	0.5 ± 0.3	—	0.3	0.8	—	—	—	—
120	781, 11.58 min	C41H66O14	—	—	781.4392	-1.5	3	6.4	3.7 ± 1.8	—	—	—	1.6	3.2	6.4	—
121	645, 11.67 min	C37H58O9	—	—	645.4039	-4.7	3	20.0	11.1 ± 6.6	12.1	1.3	20.0	—	—	—	—
122	911, 11.69 min	C47H76O17	—	—	911.5028	-2.0	2	2.6	1.7 ± 1.0	—	0.7	2.6	—	—	—	—
123	**1089.53, 11.7 min**	**C56H82O21**	927 [M−Hex−H]−<br>765 [M−Hex−Hex−H]−<br>603 [M−Hex−Hex−Hex−H]−<br>471 [M−Hex−Hex−Hex−Pen−H]−	471	1089.5320	-4.1	**6**	**33.4**	14.1 ± 10.7	20.5	2.4	**33.4**	2.0	20.5	5.9	—
124	677, 11.84 min	C36H54O12	501 [M−HexA−H]−	501	677.3561	-2.8	2	14.5	11.0 ± 3.5	—	—	—	—	14.5	7.4	—
125	1381, 11.92 min	C65H106O31	—	—	1381.6659	-1.0	2	3.4	2.5 ± 0.9	—	—	—	3.4	1.6	—	—
126	**1057, 11.92 min**	**C53H86O21**	895 (100) [M−Hex−H]−<br>733 [M−Hex−Hex−H]−<br>587 [M−Hex−Hex−dHex−H]−<br>455[M−Hex−Hex−dHex−Pen−H]−	455	1057.5601	-1.1	**4**	**42.0**	22.4 ± 14.5	—	—	—	**31.8**	8.3	7.5	**42.0**
127	663, 11.92 min	C36H56O11	—	—	663.3750	0.0	3	3.9	3.0 ± 0.9	—	—	—	1.6	3.9	3.5	—
128	1073, 12.01 min	C53H86O22	911 [M−Hex−H]−<br>765 [M−dHex−H]−<br>749 [M−Hex−H]−<br>603 [M−(Hex+dHex)−H]−<br>471 [M−(Hex+dHex)−Pen−H]−	471	1073.5571	-3.1	1	3.7	3.7 ± 0.0	—	—	—	—	—	3.7	—
129	677, 12.05 min	C36H54O12	—	—	677.3566	-3.5	2	5.0	4.5 ± 0.4	—	—	—	—	4.1	5.0	—
130	**911, 12.07 min**	**C47H76O17**	587 [M−(Hex+Hex)−H]−	—	911.5043	-3.6	**7**	**33.7**	9.8 ± 7.8	**33.7**	0.6	6.5	2.6	12.1	2.2	10.8
131	**1057, 12.18 min**	**C53H86O21**	895 (100) [M−Hex−H]−<br>733 [M−Hex−Hex−H]−<br>587 [M−Hex−Hex−dHex−H]−<br>455[M−Hex−Hex−dHex−Pen−H]−	455	1057.5600	-1.1	**7**	**25.6**	5.5 ± 5.7	1.4	0.6	0.6	5.1	3.9	1.6	**25.6**
132	1073, 12.24 min	C53H86O22	—	—	1073.5578	-3.7	1	1.9	1.9 ± 0.0	—	—	—	—	—	1.9	—
133	793, 12.35 min	C42H66O14	631 [M−Hex−H]−<br>569<br>455 [M−Hex−HexA−H]−	455	793.4380	0.0	7	17.3	4.3 ± 3.7	2.1	0.5	1.8	3.0	3.5	1.7	17.3
134	1131.54, 12.37 min	C58H84O22	—	—	1131.5396	-1.3	1	4.4	4.4 ± 0.0	—	—	4.4	—	—	—	—
135	1033-S, 12.52 min	C49H78O21S	709 [M−(Hex+Hex)−H]−<br>649 [M−(Hex+Hex)−AA−H]−<br>569 [M−(Hex+Hex)−AA−SO3−H]−<br>437 [M−(Hex+Hex)−AA−SO3−Pen−H]−	437	1033.4731	-4.6	3	8.2	5.6 ± 2.5	8.2	6.7	1.8	—	—	—	—
136	765, 12.52 min	C41H66O13	—	—	765.4460	-3.8	1	9.6	9.6 ± 0.0	—	—	—	—	—	9.6	—
137	**911, 12.6 min**	**C47H76O17**	749 [M−Hex−H]−<br>587 [M−Hex−Hex−H]−<br>455 [M−Hex−Hex−Pen−H]−	455	911.5016	-0.7	**5**	**100.0**	23.6 ± 30.6	2.2	—	1.8	—	5.7	8.2	**100.0**
138	793, 12.6 min	C42H66O14	631 [M−Hex−H]−<br>569<br>455 [M−Hex−HexA−H]−	455	793.4393	-1.7	6	12.3	6.0 ± 4.4	3.0	0.6	1.3	12.3	11.1	7.7	—
139	765, 12.66 min	C41H66O13	—	—	765.4457	-3.5	1	6.3	6.3 ± 0.0	—	—	—	—	—	6.3	—
140	1381, 12.76 min	C65H106O31	1057 [M−(Hex+Hex)−H]−<br>895 [M−(Hex+Hex)−Hex−H]−<br>733 [M−(Hex+Hex)−Hex−Hex−H]−<br>587 [M−(Hex+Hex)−Hex−Hex−dHex−H]−<br>455 [M−(Hex+Hex)−Hex−Hex−dHex−Pen−H]−	455	1381.6499	-0.5	7	24.5	6.4 ± 6.2	1.1	0.4	2.5	10.2	24.5	0.7	5.8
141	911, 12.77 min	C47H76O17	749 [M−Hex−H]−<br>587 [M−Hex−Hex−H]−<br>455 [M−Hex−Hex−Pen−H]−	455	911.5019	-1.1	6	10.1	4.7 ± 3.5	3.4	0.9	10.1	—	1.8	2.2	9.6
142	1131.54, 12.97 min	C58H84O22	—	—	1131.5409	-2.4	3	14.6	6.1 ± 5.7	2.4	1.2	14.6	—	—	—	—
143	911, 12.97 min	C47H76O17	—	—	911.5014	-0.4	1	6.5	6.5 ± 0.0	—	—	—	—	—	—	6.5
144	911, 13.12 min	C47H76O17	—	—	911.5015	-0.6	2	1.7	1.2 ± 0.6	—	0.6	—	1.7	—	—	—
145	895, 13.26 min	C47H76O16	—	—	895.5084	-2.7	1	2.0	2.0 ± 0.0	—	—	—	2.0	—	—	—
146	1131.54, 13.33 min	C58H84O22	—	—	1131.5397	-1.3	1	1.9	1.9 ± 0.0	—	—	1.9	—	—	—	—
147	1115, 13.37 min	C55H88O23	—	—	1115.5631	1.1	1	2.0	2.0 ± 0.0	—	—	—	—	—	—	2.0
148	1235.58, 13.65 min	C62H92O25	911 [M−(Hex+Hex)−H]−<br>587 [M−(Hex+Hex)−(Hex+Hex)−H]−<br>587 [M−(Hex+Hex)−(Hex+Hex)−H]−<br>455 [M−(Hex+Hex)−(Hex+Hex)−Pen−H]−	455	1235.5874	-1.5	1	3.6	3.6 ± 0.0	—	—	3.6	—	—	—	—
149	1085, 13.71 min	C54H86O22	881 [M−(Hex+Ac)−H]−<br>749 [M−(Hex+Ac)−Pen−H]−<br>587 [M−(Hex+Ac)−Pen−Hex−H]−<br>455 [M−(Hex+Ac)−Pen−Hex−Pen−H]−	455	1085.5536	0.2	1	4.2	4.2 ± 0.0	—	—	—	—	—	—	4.2
150	1131.54, 13.9 min	C58H84O22	969 [M−Hex−H]−	—	1131.5406	-2.2	2	8.4	4.9 ± 3.5	1.4	—	8.4	—	—	—	—
151	**911, 13.98 min**	**C47H76O17**	749 [M−Hex−H]−<br>765 [M−dHex−H]−<br>603 [M−(Hex+dHex)−H]−<br>471 [M−(Hex+dHex)−Pen−H]−	471	911.5051	-4.5	**6**	**100.0**	**39.8 ± 37.0**	6.5	1.7	—	**38.1**	**90.6**	**100.0**	1.8
152	1131.54, 14.01 min	C58H84O22	—	—	1131.5402	-1.8	2	1.1	0.9 ± 0.2	0.7	—	1.1	—	—	—	—
153	953, 14.14 min	C49H78O18	911 [M−Ac−H]−<br>749 [M−Ac−Hex−H]−<br>731 [M−Ac−Hex−H2O−H]−<br>629 [M−(Hex+Hex)−H]−<br>587 [M−Ac−Hex−Hex−H]−<br>455 [M−(Hex+Ac)−Hex−Pen−H]−	455	953.5123	-0.8	3	3.1	1.7 ± 1.0	1.9	0.3	3.1	—	—	—	—
154	663, 14.18 min	C36H56O11	487 [M−HexA−H]−	487	663.3760	-1.5	4	11.8	7.1 ± 4.2	—	—	—	11.8	10.8	5.2	0.7
155	**911, 14.25 min**	**C47H76O17**	749 [M−Hex−H]−<br>765 [M−dHex−H]−<br>603 [M−(Hex+dHex)−H]−<br>471 [M−(Hex+dHex)−Pen−H]−	471	911.5051	-4.5	**7**	**53.4**	17.4 ± 14.4	9.1	6.4	1.8	17.1	**31.7**	**53.4**	2.2
156	895, 14.43 min	C47H76O16	733 [M−Hex−Hex−H]−<br>587 [M−Hex−Hex−dHex−H]−<br>455[M−Hex−Hex−dHex−Pen−H]−	455	895.5077	-1.8	1	20.6	20.6 ± 0.0	—	—	—	—	—	—	20.6
157	809, 14.68 min	C42H66O15	765 [M−CO2−H]−<br>471 [M−CO2−(Hex+Pen)−H]−	471	809.4358	-3.6	1	8.3	8.3 ± 0.0	—	—	—	—	—	8.3	—
158	895, 14.71 min	C47H76O16	733 [M−Hex−Hex−H]−<br>587 [M−Hex−Hex−dHex−H]−	—	895.5069	-1.0	3	7.8	3.8 ± 2.7	—	—	—	—	2.2	1.3	7.8
159	**953, 14.96 min**	**C49H78O18**	749 [M−(Hex+Ac)−H]−<br>731 [M−(Hex+Ac)−H2O−H]−<br>587 [M−(Hex+Ac)−Hex−H]−<br>455 [M−(Hex+Ac)−Hex−Pen−H]−	455	953.5118	-0.3	**1**	**31.8**	**31.8 ± 0.0**	—	—	—	—	—	—	**31.8**
160	**765, 15.25 min**	**C41H66O13**	603 [M−Hex−H]−<br>471 [M−Hex−Pen−H]−	471	765.4459	-3.6	**6**	**93.8**	**28.9 ± 31.2**	12.0	1.4	1.0	7.6	**57.7**	**93.8**	—
161	765, 15.52 min	C41H66O13	603 [M−Hex−H]−	—	765.4455	-3.1	6	22.5	6.7 ± 6.3	3.2	1.2	1.7	1.9	9.9	22.5	—
162	911, 15.76 min	C47H76O17	—	—	911.5046	-3.9	2	8.8	6.9 ± 1.8	—	—	—	8.8	—	5.1	—
163	871-S, 15.79 min	C43H68O16S	709 [M−Hex−H]−<br>649 [M−Hex−AA−H]−<br>569 [M−Hex−AA−SO3−H]−<br>437 [M−Hex−AA−SO3−Pen−H]−	437	871.4155	0.0	1	0.9	0.9 ± 0.0	—	0.9	—	—	—	—	—
164	911, 16.03 min	C47H76O17	—	—	911.5054	-4.9	2	7.7	5.2 ± 2.5	—	—	—	2.7	—	7.7	—
165	**953, 16.33 min**	**C49H78O18**	629 [M−(Hex+Hex)−H]−	—	953.5165	-5.2	**3**	**26.6**	11.8 ± 9.9	**26.6**	0.2	8.7	—	—	—	—
166	1219.59, 16.36 min	C62H92O24	1057 [M−Hex−H]−<br>895 [M−Hex−Caf−H]−<br>733 [M−Hex−Caf−Hex−H]−<br>597 [M−Hex−Caf−Hex−dHex−H]−<br>455 [M−Hex−Caf−Hex−dHex−Pen−H]−	455	1219.5903	0.2	2	8.9	7.0 ± 1.9	—	—	—	5.1	—	—	8.9
167	**647, 16.5 min**	**C36H56O10**	471 [M−HexA−H]−	471	647.3830	-4.6	**6**	**43.1**	16.4 ± 17.0	3.4	0.6	0.2	10.6	**40.7**	**43.1**	—
168	1219.59, 16.65 min	C62H92O24	1057 [M−Hex−H]−<br>895 [M−Hex−Caf−H]−<br>733 [M−Hex−Caf−Hex−H]−<br>597 [M−Hex−Caf−Hex−dHex−H]−<br>455 [M−Hex−Caf−Hex−dHex−Pen−H]−	455	1219.5909	-0.3	2	7.0	4.1 ± 2.9	—	—	—	1.1	—	—	7.0
169	807, 16.84 min	C43H68O14	747 [M−H2O−H]−<br>603 [M−Hex−H]−<br>471 [M−Hex−Pen−H]−	471	807.4536	0.1	3	3.3	2.0 ± 0.9	3.3	1.8	0.8	—	—	—	—
170	807, 17.08 min	C43H68O14	747 [M−H2O−H]−<br>603 [M−Hex−H]−<br>471 [M−Hex−Pen−H]−	471	807.4539	-0.4	3	1.8	1.6 ± 0.2	1.2	1.7	1.8	—	—	—	—
171	**749, 17.18 min**	**C41H66O12**	453 [M−Cou−Pen−H]−	453	745.4515	-4.5	**5**	**27.6**	10.1 ± 11.4	1.0	0.2	—	0.6	21.0	**27.6**	—
172	871-S, 17.22 min	C43H68O16S	709 [M−Hex−H]−<br>649 [M−Hex−AA−H]−<br>569 [M−Hex−AA−SO3−H]−<br>437 [M−Hex−AA−SO3−Pen−H]−	437	871.4165	-1.1	4	6.1	3.2 ± 2.1	6.1	2.0	0.3	—	4.4	—	—
173	749, 17.43 min	C41H66O12	—	—	745.4511	-3.9	4	11.3	4.6 ± 3.8	1.3	0.3	—	—	5.6	11.3	—
174	749, 18.3 min	C41H66O12	471 [M−(Pen+dHex)−H]−	471	749.4514	-4.3	1	6.6	6.6 ± 0.0	—	—	—	—	—	6.6	—
175	749, 18.58 min	C41H66O12	471 [M−(Pen+dHex)−H]−	471	749.4518	-4.9	1	17.5	17.5 ± 0.0	—	—	—	—	—	17.5	—
176	807, 18.73 min	C43H68O14	—	—	807.4542	-0.7	3	2.0	1.3 ± 0.5	2.0	0.6	1.4	—	—	—	—
177	895, 20.49 min	C47H76O16	733 [M−Hex−Hex−H]−<br>587 [M−Hex−Hex−dHex−H]−<br>455[M−Hex−Hex−dHex−Pen−H]−	455	895.5074	-1.5	4	11.4	8.0 ± 1.7	—	—	—	11.4	7.4	7.7	5.3
178	895, 20.61 min	C47H76O16	—	—	895.5050	1.2	4	6.0	5.0 ± 0.9	—	—	—	4.1	6.0	5.8	4.2
179	749, 22.28 min	C41H66O12	587 [M−Hex−H]−<br>455 [M−Hex−Pen−H]−	455	749.4506	-3.2	2	11.8	10.6 ± 1.2	—	—	—	—	—	11.8	9.4

Samples abbreviations: *Ilex aquifolium* cultivars (Aurea Marginata – AM, Silver Queen – SQ, Handsworth New Silver – HNS), *Ilex × meserveae* cultivars (Blue Prince – BP, Blue Princess– BPR, and Blue Girl – BG), reference *Ilex paraguariensis* (sample of Yerba Mate - PA ref.).
RT—retention time, RA %—the relative area of peak, when the area of the largest one in the sample is calculated as 100%.
MS/MS losses abbreviations: Ac—acetyl, AA—acetic acid, Cou—coumaric acid, dHex—deoxyhexose, Hex—hexose, HexA—hexuronic acid, Pen—pentose.
Neutral formula equivalents for aglycone [M]⁻: 437—C30H46O2, 439—C30H48O2, 453—C30H46O3, 455—C30H48O3, 471—C30H48O4, 487—C30H48O5, 501—C30H46O6.
Highlighting: bold font—saponin with RA over 25%, **dark grey**—saponin marker for *I. paraquariensis* reference, **green**—saponin marker for *I. aquifolium* group, **red**—saponin marker for *I. meserveae* group.
