# Supplementary figures and images for: Antiviral and Cytotoxic Activities of Ilex aquifolium Silver Queen in the Context of Chemical Profiling of Two Ilex Species
Source: Molecules. 2024 Jul 8;29(13):3231. doi: 10.3390/molecules29133231 (PMC11243556; doi:10.3390/molecules29133231)

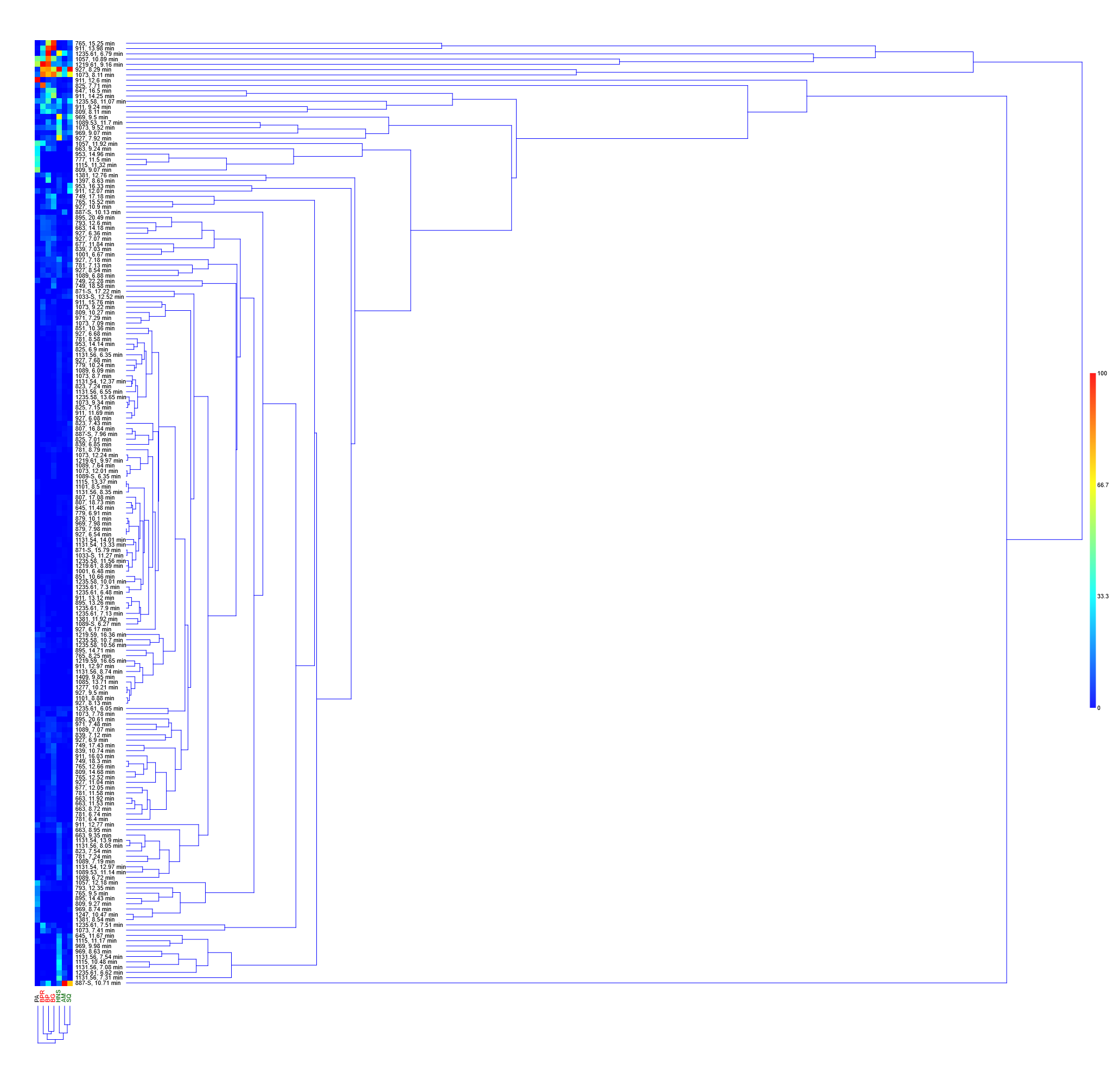

Supplement: Supplementary file 1 [file molecules-29-03231-s001.zip › Fig S1.png]
